# Phosphorylated Histone 3 at Serine 10 Identifies Activated Spinal Neurons and Contributes to the Development of Tissue Injury-Associated Pain

**DOI:** 10.1038/srep41221

**Published:** 2017-01-25

**Authors:** Jose Vicente Torres-Pérez, Péter Sántha, Angelika Varga, Peter Szucs, Joao Sousa-Valente, Botond Gaal, Miklós Sivadó, Anna P Andreou, Sara Beattie, Bence Nagy, Klara Matesz, J. Simon C. Arthur, Gábor Jancsó, Istvan Nagy

**Affiliations:** 1 Nociception Group, Section of Anaesthetics, Pain Medicine and Intensive Care, Department of Surgery and Cancer, Imperial College London, London, SW10 9NH, United Kingdom; 2 Department of Physiology, University of Szeged, Szeged, H-6720, Hungary; 3 MTA-DE-NAP B-Pain Control Research Group, University of Debrecen, Debrecen, H-4012, Hungary; 4 Department of Anatomy, Histology and Embryology, University of Debrecen, Debrecen, H-4012, Hungary; 5 The Ipswich Hospital, Ipswich, IP4 5PD, United Kingdom; 6 Division of Cell Signalling and Immunology, College of Life Sciences, Sir James Black Centre, University of Dundee, Dundee DD1 5EH, United Kingdom

## Abstract

Transcriptional changes in superficial spinal dorsal horn neurons (SSDHN) are essential in the development and maintenance of prolonged pain. Epigenetic mechanisms including post-translational modifications in histones are pivotal in regulating transcription. Here, we report that phosphorylation of serine 10 (S10) in histone 3 (H3) specifically occurs in a group of rat SSDHN following the activation of nociceptive primary sensory neurons by burn injury, capsaicin application or sustained electrical activation of nociceptive primary sensory nerve fibres. In contrast, brief thermal or mechanical nociceptive stimuli, which fail to induce tissue injury or inflammation, do not produce the same effect. Blocking N-methyl-D-aspartate receptors or activation of extracellular signal-regulated kinases 1 and 2, or blocking or deleting the mitogen- and stress-activated kinases 1 and 2 (MSK1/2), which phosphorylate S10 in H3, inhibit up-regulation in phosphorylated S10 in H3 (*p*-S10H3) as well as *fos* transcription, a down-stream effect of *p*-S10H3. Deleting MSK1/2 also inhibits the development of carrageenan-induced inflammatory heat hyperalgesia in mice. We propose that *p*-S10H3 is a novel marker for nociceptive processing in SSDHN with high relevance to transcriptional changes and the development of prolonged pain.

Prolonged pain presents a major unmet clinical need because the currently available analgesics, due to their low efficacy, often provide unsatisfactory pain relief^1^. Attempts to translate promising candidate molecules into clinically useful effective analgesics have repeatedly failed in the last three decades. Therefore, in order to identify targets for the development of novel analgesics to improve the relief of prolonged pain, alternative approaches to elucidate fundamental mechanisms must be employed.

The development of prolonged pain depends on long-term increase in the efficacy of nociceptive signal transduction, which includes activity-dependent increase in the excitability and activity (sensitisation) of, and altered synaptic strength between, neurons involved in nociceptive processing^2^,^3^. Although sensitisation occurs at all levels of the pain-processing pathways^4^, this process is particularly important in the superficial spinal dorsal horn, because neuronal circuitries in that area form the “gateway” of nociceptive information towards supraspinal centres where the pain experience manifests^5^.

Sensitisation including that of spinal cord neurons (spinal sensitisation) involves activity-dependent post-translational changes in membrane molecules and changes in gene transcription which respectively occur minutes and tens of minutes – hours following the spinal nociceptive input^2^,^3^,^4^,^6^. Alterations in gene expression are particularly important for the maintenance of sensitisation^2^,^3^,^4^,^6^.

Epigenetic mechanisms including post-translational modifications (PTM) in histones, which occur as specific responses to various changes in the cellular environment, regulate transcription through the alteration of access to the DNA for proteins involved in gene expression^7^. Histone 3 (H3) is one type of the histone isoforms, which compile the octomeric protein core of nucleosomes^7^,^8^. Similarly to other histones, H3 also hosts a series of consensus sites for different types of PTM including phosphorylation^7^,^8^.

In addition to neurons involved in nociceptive processing, neurons involved in other functions of the nervous system, including the formation of memory in the hippocampus also exhibit activity-dependent increase in the efficacy of signal transduction, which is referred to as long term potentiation (LTP)^2^. Importantly, spinal sensitisation and LTP share a series of similarities in their respective cellular and molecular mechanisms including their dependence on transcriptional changes^2^. Phosphorylation of serine 10 (S10) in H3 in the hippocampus has recently been associated with chromatin re-modelling and subsequent changes in gene transcription and synaptic connections as well as alterations in behaviour following environmental challenges^9^,^10^,^11^,^12^. Here, we studied whether phosphorylated (*p*) S10 in H3 (*p*-S10H3) is associated with transcriptional changes in superficial spinal dorsal horn neurons (SSDHN) and subsequent alterations in behaviour. We report that tissue injury, inflammation and persistent, but not brief non-tissue-damaging noxious stimuli specifically phosphorylate S10 in H3, in a group of SSDHN and that the phosphorylation is mediated through the N-methyl-D-aspartate (NMDA) receptor – extracellular signal-regulated kinases 1 and 2 (ERK1/2) – mitogen and stress activated kinases 1 and 2 (MSK1/2) pathways. We further report that blocking S10H3 phosphorylation results in the lack of expression of c-Fos, a *p*-S10H3 down-stream target that contributes to the development of pain^13^,^14^,^15^,^16^,^17^,^18^. During the revision of this manuscript Tochiki and co-workers have reported that *p*-S10H3 contributes to the development of formalin injection-evoked pain-related behaviour^19^.

## Results

### Burn injury up-regulates *p*-S10H3 expression in a sub-population of SSDHN

Burn injury is a frequent traumatic tissue injury followed by an inflammatory reaction and excruciating pain^20^. Therefore, first we assessed *p*-S10H3 expression in the spinal dorsal horn of rats following the induction of burn injury according to our established experimental model^21^.

**Figure 1:**
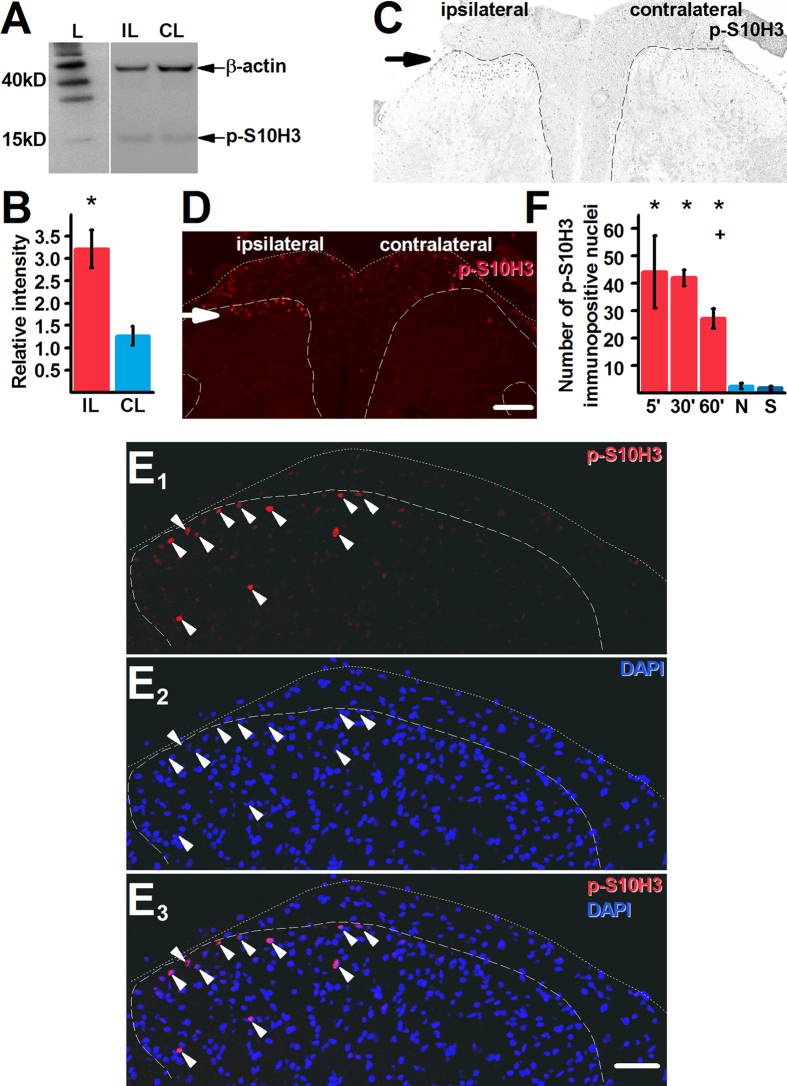
Burn injury induces prolonged up-regulation in *p*-S10H3 expression in superficial spinal dorsal horn of the spinal cord. (**A**) and (**B**) Burn injury increases *p*-S10H3 (expected MW: 17kD) expression within 5 minutes in the ipsilateral side (IL) compared to the contralateral side (CL). The expected molecular weight of β-actin is 42 kD. L indicates size marker. *p = 0.006; n = 4. The Western blot images shown in (**A**) are cropped. (**C**) and (**D**) Images of transverse sections of the L4-L5 spinal segments showing both the ipsilateral and contralateral sides. *p*-S10H3-immunopositive structures in the ipsilateral spinal cord (arrow; scale bar = 200 μm). (E_1_–E_3_) Images of the ipsilateral side of a transverse spinal cord section of the L4-L5 segments. Structures exhibiting *p*-S10H3-immunopositivity in the superficial spinal dorsal horn are invariably positive also to DAPI which binds to adenine-thymine regions of the DNA in the nucleus. Scale bar = 100 μm. (**F**) *p*-S10H3 expression lasts for at least 60 minutes (60′) after the injury. 5′, 30′ and 60′, 5, 30 and 60 minutes post-injury, respectively; N, naive; S, 5 minutes after a sham injury. *p < 0.001, + p = 0.001 (n = 4). Dotted and dashed lines indicate the surface of the spinal cord and the white-grey matter border, respectively on each image.

Western-blotting and subsequent densitometric analysis revealed that burn injury significantly up-regulated *p*-S10H3 expression (expected molecular weight is 17 kD) in the ipsilateral spinal dorsal horn (Fig. 1A and B; p = 0.006, Student’s t-test) 5 minutes after the injury. Immunohistochemistry confirmed the up-regulation of *p*-S10H3 expression in the spinal cord (Fig. 1C and D). The overwhelming majority of the *p*-S10H3-immunopositive profiles distributed in the superficial laminae of the ipsilateral spinal dorsal horn (Fig. 1C and D) and exhibited immunolabelling with the fluorescent dye 4′, 6-diamidino-2-phenylindole (DAPI) which binds to adenine-thymine rich regions in DNA^22^ (Fig. 1E1–E_3_). Hence, we regarded *p*-S10H3-immunolabelled structures as nuclei.

On average, the total number of *p*-S10H3-immunopositive nuclei, in the L4-L5 ipsilateral spinal dorsal horn of 6 sections, each of which were 100 μm apart from the adjacent neighbours (see Methods), was ~45 (44.25 ± 13.33, n = 4). The number of nuclei found in the ipsilateral side was significantly (p < 0.001, GLM) more than in the spinal dorsal horn of naive (Fig. 1F; 2.50 ± 1.04, n = 4) or sham animals (Fig. 1F; 1.75 ± 0.63, n = 4), or in the contralateral side of the spinal cord in injured rats (3 ± 3, n = 4; Fig. 1C,D; Supplementary Figure 1). The burn injury-induced up-regulation in *p*-S10H3 expression was evident also at 30 and 60 minutes after the injury (the longest survival time we examined), though at 60 minutes, an ~30%, significant (p = 0.001, GLM) reduction in the number of *p*-S10H3-immunopositive nuclei was found (27.25 ± 3.52, n = 4; Fig. 1F).

**Figure 2:**
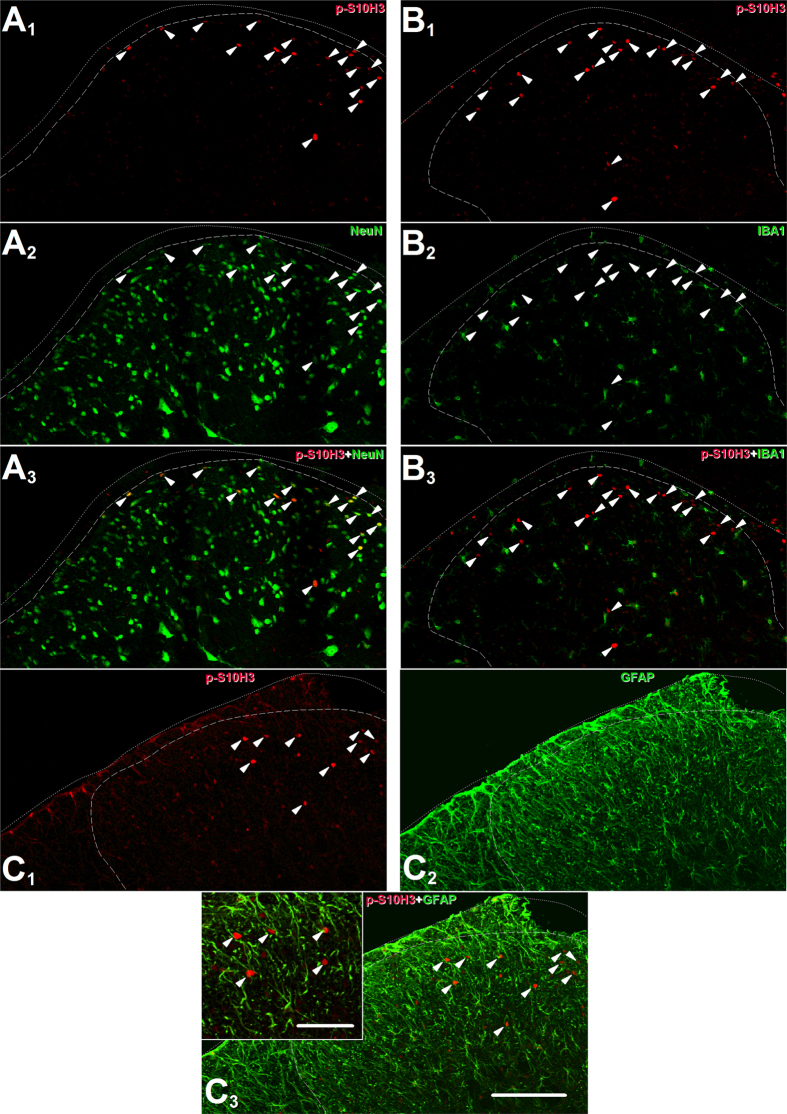
The burn injury-induced up-regulation in *p*-S10H3 expression occurs in neurons. (**A**_**1–3**_) Images of the ipsilateral side of a section cut from the L4-L5 spinal segments. *p*-S10H3-expressing structures (arrows) 5 minutes after burn injury also express the neuronal nucleus marker NeuN. (**B**_**1–3**_) Images of the ipsilateral side of a section cut from the L4-L5 spinal segments. Microglia identified with an anti-ionised calcium-binding adaptor protein 1 (IBA1) antibody do not express *p*-S10H3 after burn injury. (**C_1–3_**) Images of the ipsilateral side of a section cut from the L4-L5 spinal segments. Astrocytes identified with an anti-glial fibrillary acidic protein (GFAP) antibody do not express *p*-S10H3 after burn injury. (Scale bar, 200 μm; scale bar in inset, 100 μm.) Dotted and dashed lines indicate the surface of the spinal cord and the white-grey matter border on each image.

Combined staining with the neuron specific anti-hexaribonucleotide-binding protein-3 (NeuN) antibody, the microglia specific anti-complement receptor 3 (CD11b) antibody or the astrocyte specific anti-glial fibrillary acidic protein (GFAP) antibody showed that *p*-S10H3 is expressed only in neurons following burn injury, because all *p*-S10H3-immunopositive nuclei also expressed NeuN, whereas none of them was surrounded by CD11b- or GFAP-immunolabelled cytoplasm (Fig. 2A1–3–C_1–3_).

### SSDHN exhibiting *p*-S10H3-immunopositivity in the nucleus are in close apposition to spinal terminals of nociceptive primary sensory neurons

**Figure 3:**
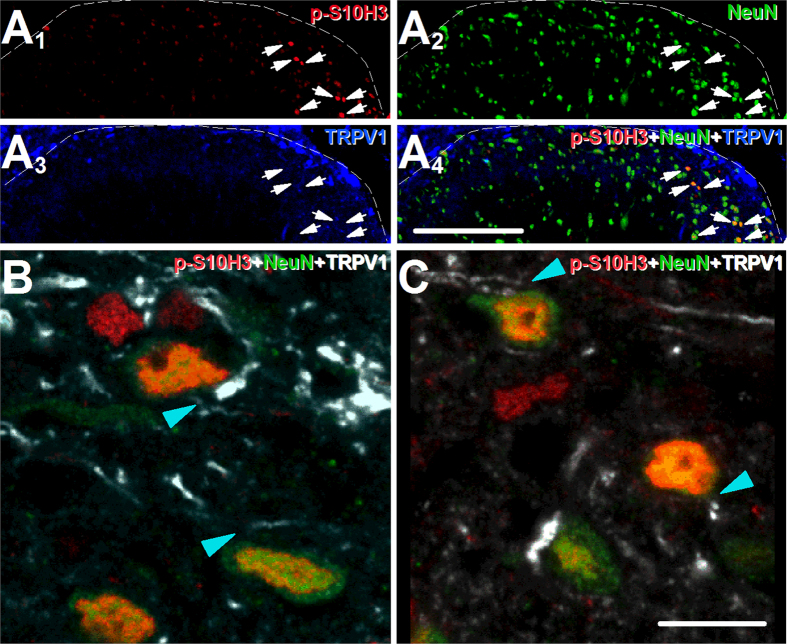
*p*-S10H3 expression occurs in SSDHN, which are in close apposition to spinal terminals of nociceptive primary sensory neurons. (**A**_**1**_–**A**_**4**_) Images of the ipsilateral side of a section cut from the L4-L5 spinal segments. *p*-S10H3-expressing nuclei (arrows), 5 minutes after burn injury are distributed among primary sensory nerve fibres expressing TRPV1. (Scale bar, 400 μm). (**B**) and (**C**) Images of single optical sections of 2 μm thickness of the spinal dorsal horn 5 minutes following burn injury. Virtually, all neurons expressing *p*-S10H3 in the nucleus, 5 minutes after burn injury are in close apposition to TRPV1-expressing nerve fibres (arrow heads). (Scale bar, 10 μm). Dashed lines indicate the white-grey matter border on each image.

The great majority of nociceptive primary sensory neurons express the transient receptor potential cation channel sub-family V member 1 ion channel (TRPV1) that is pivotal in signalling, to the central nervous system, inflammatory events in peripheral tissues that quickly develop following burn injury^20^,^23^,^24^,^25^. Therefore, we studied the relationship of TRPV1-expressing spinal terminals of nociceptive primary sensory neurons and SSDHN exhibiting *p*-S10H3 expression in the nucleus (Fig. 3)^26^,^27^. We found that SSDHN with *p*-S10H3-immunopositive nucleus were distributed among TRPV1-immunopositive terminals (Figure 3A1–A_4_). Further, the great majority of SSDHN exhibiting *p*-S10H3-immunopositive nucleus were in close apposition to TRPV1-immunolabelled structures (Fig. 3B and C).

### *p*-S10H3 is co-expressed with phosphorylated extracellular signal-regulated kinase 1 and 2 (*p*-ERK1/2) and c-Fos

**Figure 4:**
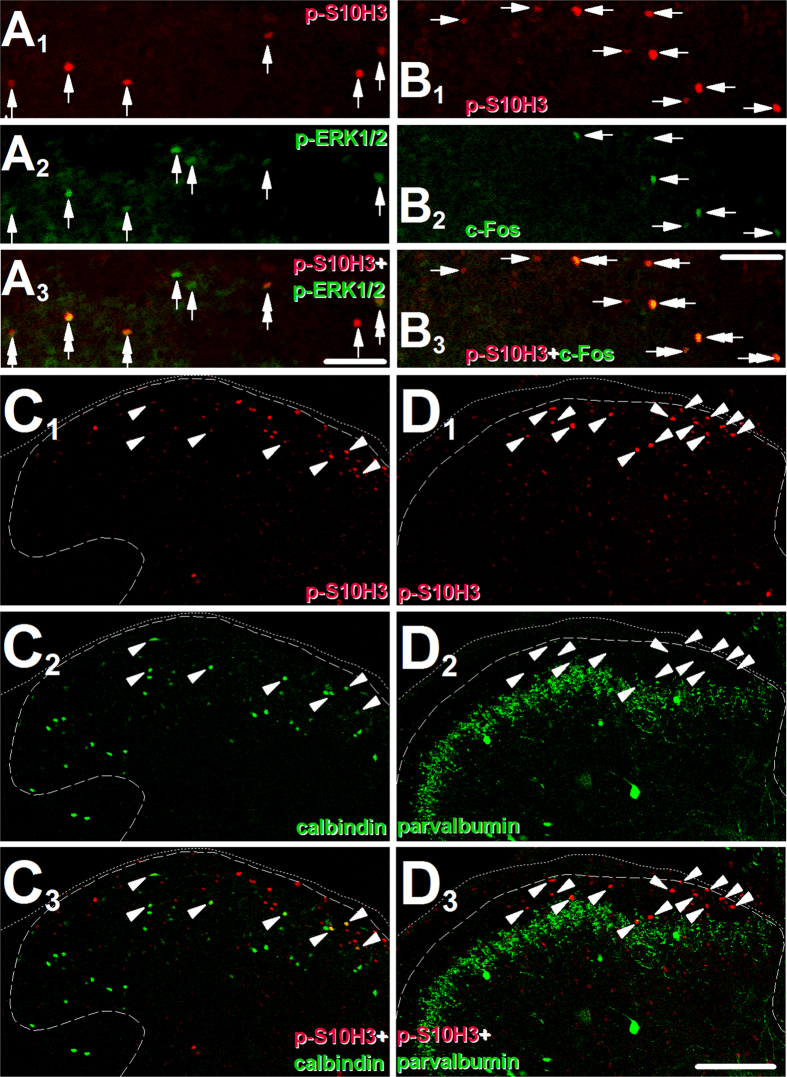
The majority of SSDHN expressing *p*-S10H3 following burn injury are excitatory neurons and also express *p*-ERK1/2 or c-Fos. (**A**_**1**_–**A**_**3**_) Images of the ipsilateral side of a section cut from the L4-L5 spinal segments. The great majority of *p*-S10H3-immunopositive nuclei (indicated by arrows in A_1_) also express *p*-ERK1/2 (double arrows in A_3_), 5 minutes after burn injury. Arrows in A_2_ indicate neurons expressing *p*-ERK1/2, arrows in A_3_ indicate neurons expressing either *p*-S10H3 or *p*-ERK1/2 alone. (Scale bar, 50 μm). (**B**_**1**_–**B**_**3**_) Images of the ipsilateral side of a section cut from the L4-L5 spinal segments. The great majority of neurons with *p*-S10H3-immunopositive nuclei (indicated by arrows in B_1_) also express c-Fos (double arrows in B_2_ and B_3_) 30 minutes after burn injury. Arrows in B_2_ indicate neurons expressing c-Fos, arrows in B_3_ indicate neurons expressing either c-Fos or *p*-ERK1/2 alone. (Scale bar, 50 μm). (**C**_**1**_–**C**_**3**_) Images of the ipsilateral side of a section cut from the L4-L5 spinal segments. A major proportion of neurons with *p*-S10H3-immunopositive nucleus also express calbindin, a marker of a sub-population of excitatory SSDHN. Arrowheads indicate double-labelled neurons in all images. (Scale bar, 200 μm). (**D**_**1**_–**D**_**3**_) Images of the ipsilateral side of a section cut from the L4-L5 spinal segments. Neurons with *p*-S10H3-immunopositive nucleus were never seen to express parvalbumin, a marker of a sub-population of inhibitory SSDHN. Arrowheads indicate neurons expressing *p*-S10H3 or the location of neurons in **D**_**2**_. (Scale bar, 200 μm). Dotted and dashed lines indicate the surface of the spinal cord and the white-grey matter border, respectively on each image.

Burn injury, up-regulates the expression of *p*-ERK1/2 in a group of SSDHN within 5 minutes after the injury^21^. Nociceptive stimuli also up-regulate the expression of c-Fos, the protein product of the immediate early gene *fos*^17^. We found that, at 5 minutes post-injury time, 57 ± 8.12% (n = 4) of the *p*-S10H3-immunopositive nuclei were also immunopositive for *p*-ERK1/2, whereas 84.74 ± 1.85% (n = 4) of the *p*-ERK1/2 immunopositive nuclei exhibited *p*-S10H3 immunolabelling (Fig. 4A1–A_3_). At 30 minutes post-injury time, 72.2 ± 7.73% (n = 4) of the *p*-S10H3-immunopositive nuclei also expressed c-Fos, whereas 66.98 ± 14.54% (n = 4) of the c-Fos-immunopositive nuclei exhibited *p*-S10H3-immunolabelling (Fig. 4B1–B_3_).

### *p*-S10H3 expression appears in excitatory rather than inhibitory neurons

Neurons in the spinal dorsal horn are either excitatory or inhibitory in function and they can be identified, in addition to their effect on responses of other neurons by their neurochemical properties^5^. Sub-populations of inhibitory and excitatory spinal dorsal horn neurons respectively express parvalbumin and calbindin^5^. Here, we found that, 5 minutes after burn injury *p*-S10H3 was never co-expressed with parvalbumin, whereas several calbindin-expressing cells exhibited *p*-S10H3 immunostained nuclei (Fig. 4C_1_–C_3_ and D_1_–D_3_).

### Capsaicin application to the hind paw up-regulates *p*-S10H3 expression in SSDHN

Topical application or injection of capsaicin, the archetypal TRPV1 agonist^25^ induces, without producing tissue damage, an inflammatory response^24^,^25^. The inflammatory response is accompanied by sensitisation of spinal dorsal horn neurons and subsequent development of prolonged pain^28^.

**Figure 5:**
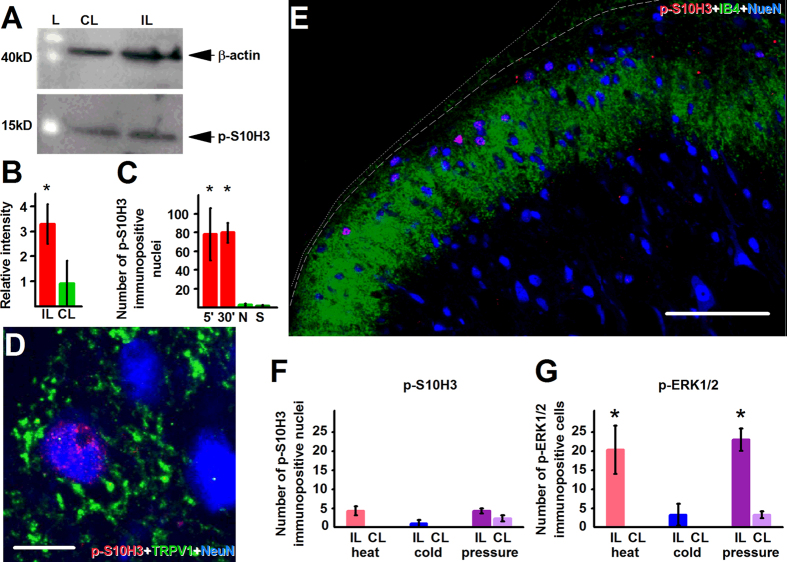
Capsaicin application does, whereas brief painful stimuli which do not damage tissues do not, induce *p*-S10H3 expression in SSDHN. (**A**) and (**B**) Capsaicin injection increases *p*-S10H3 expression (expected MW: 17 kD) in the ipsilateral (IL) spinal cord within 5 minutes. CL, contralateral; L indicates size marker, *p < 0.04, n = 6. The expected molecular weight of β-actin is 42 kD. The Western blot images shown in (**A**) are cropped. (**C**) The ipsilateral L4-L5 spinal cord exhibits a significant increase in the number of neurons with *p*-S10H3-expressing nuclei 5 (5’) and 30 (30’) minutes after capsaicin injection. N, “ipsilateral” side of naive; S, “ipsilateral” side of saline-injection. *p < 0.001, n = 4. (**D**) *p*-S10H3-immunopositive nucleus-exhibiting neurons, following capsaicin injection are often seen in close apposition to TRPV1-expressing primary sensory neuron terminal-like profiles. Single optical section of 2.2 μm. (Scale bar, 10 μm). (**E**) An image of the ipsilateral side of a section cut from the L4-L5 spinal segments. Topical application of capsaicin onto the paw also induces p-S10H3 expression in the superficial laminae of the spinal cord. IB4-staining was used to indicate the ventral border of lamina II_i_. The dotted and dashed lines indicate the surface of the spinal cord and the white-grey matter border, respectively. (Scale bar, 100 μm). (**F**) Quantification of *p*-S10H3 immunolabelled nuclei in the ipsilateral (IL) and contralateral (CL) superficial spinal dorsal horn 5 minutes after repeated noxious heat (44 °C), noxious cold (4 °C) or noxious pressure (6 N/cm^2^) stimulation. n = 3. (**H**) Quantification of *p*-ERK1/2-immunolabelled nuclei in the ipsilateral (IL) and contralateral (CL) superficial spinal dorsal horn 5 minutes after repeated noxious heat (44 °C), noxious cold (4 °C) or noxious pressure (6 N/cm^2^) stimulation. *p < 0.001, n = 3.

Western-blotting showed that capsaicin injection increased *p*-S10H3 expression in the spinal cord 5 minutes after the injection (p = 0.04, Student’s t-test; Fig. 5A and B). Capsaicin injection into the paw also significantly increased the number of nuclei expressing *p*-S10H3-immunolabelling in a group of SSDHN within 5 minutes (78.33 ± 27.79, (n = 3) after the injection; 3.33 ± 0.88 in naive (n = 3); p < 0.001, GLM; Fig. 5C and Supplementary Figure 2A). The up-regulation persisted for at least 30 minutes, the longest survival time we examined after the injection (80.00 ± 10.44, n = 3; Fig. 5C and Supplementary Figure 2B). The contralateral spinal cord did not exhibit similar increase in *p*-S10H3 expression (Supplementary Figure 3). Saline injection did not change the number of *p*-S10H3-immunopositive nuclei either in the ipsilateral (2.00 ± 1.00, n = 3; p = 0.914 GLM, Fig. 5C) or contralateral side (data not shown). Again, nuclei, expressing *p*-S10H3-immunopositivity after capsaicin injection belonged to neurons because cells, which expressed such nuclei, also expressed NeuN but were not surrounded by CD11b- or GFAP-immunolabelled cytoplasm (data not shown). Further, ~3/4 of SSDHN, which exhibited *p*-S10H3-immunolabeled nuclei (74.43 ± 0.98%, n = 3), appeared in close apposition to TRPV1-immunolabelled fibres/terminals 5 minutes following capsaicin application (Fig. 5D). In addition to capsaicin injection, activation of epidermal nociceptive afferents with topical application of capsaicin also induced S10H3 phosphorylation in a group of SSDHN (Fig. 5E).

### Brief noxious stimuli, which fail to induce tissue injury, do not up-regulate *p*-S10H3 expression in SSDHN

In contrast to burn injury and capsaicin application, exposure of the paw to 44 °C^29^ for 10 seconds repeatedly in every 20 seconds for 2 minutes (to avoid tissue damage) failed to induce *p*-S10H3 up-regulation (4.33 ± 1.2, n = 3; p = 0.351, GLM; Fig. 5F) whereas it significantly increased *p*-ERK1/2 expression (Fig. 5G). Exposure of the paw to 4 °C water or to 600 mN/mm^2^ pressure in a similar fashion did not induce *p*-S10H3 expression either (noxious cold: 1.00 ± 1.00, n = 3; p = 0.689, GLM; noxious pressure: 4.33 ± 0.06, n = 3, p = 0.281, GLM, Fig. 5F). While noxious cold did not, noxious pressure did increase the number of neurons expressing *p*-ERK1/2 (noxious cold: 3.33 ± 0.88, n = 3; noxious pressure: 23.00 ± 2.88, n = 3; p < 0.001, GLM, Fig. 5G).

### High frequency electrical stimulation of nociceptive unmyelinated C-fibres, but not low frequency electrical stimulation of myelinated Aβ and Aδ-fibres up-regulates *p*-S10H3 expression in SSDHN

**Figure 6:**
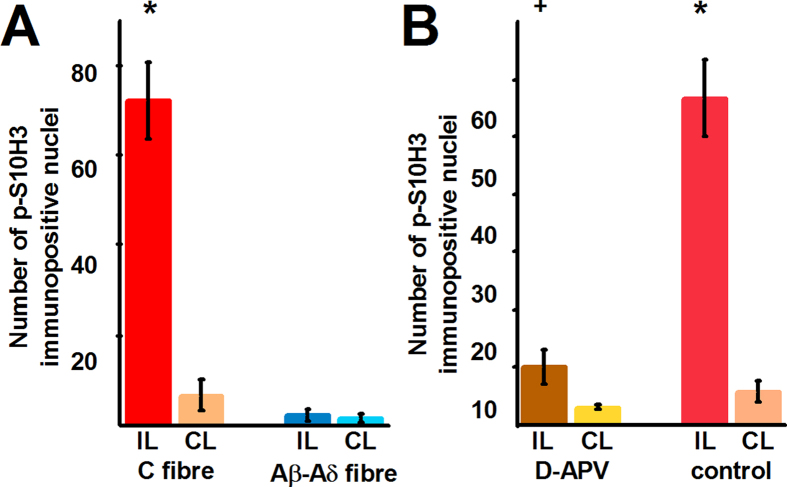
Repetitive electrical stimulation of nociceptive primary sensory neurons up-regulates *p*-S10H3 expression, in an NMDA receptor-dependent manner, in a group of SSDHN. (**A**) Bar chart showing the number of *p*-S10H3-immunopositive nuclei in the ipsilateral (IL) and contralateral (CL) dorsal horn of the spinal cord after repetitive, C fibre strength (10 trains of 200 pulses of 30 V amplitude and 2 ms width at 10 Hz frequency in every 30 second for 5 minutes) and Aβ and Aδ fibre strength (10 trains of 2 pulses of 15 V amplitude and 100 μs width with 0.1 Hz frequency in every 30 second for 5 minutes) electrical stimulation of the femoral nerve. *p < 0.001, n = 3. (**B**) Bar chart showing the number of *p*-S10H3-immunopositive nuclei in the ipsilateral (IL) and contralateral (CL) dorsal horn of *in vitro* spinal cord preparations after the L4 and L5 dorsal roots were stimulated with electrical pulses with C fibre strength (500 μA in amplitude and 1 ms in width were delivered at 2 Hz for 15 seconds, followed by 15 seconds without stimulation) either in control bath solution (control) or in the presence of the NMDA receptor antagonist D-APV (50 μM; D-APV). *p < 0.001, n = 4; + p < 0.001, n = 4.

Repetitive electrical stimulation of primary sensory neurons, similarly to activation of those cells by capsaicin, noxious heat or inflammatory mediators, induces transmitter release from the spinal terminals of those neurons. Five minutes high frequency (10 Hz) electrical stimulation of primary afferent fibres including nociceptive C-type fibres induced up-regulation in *p*-S10H3 expression in the ipsilateral superficial spinal dorsal horn (Supplementary Figure 4A and Fig. 6A). The number of *p*-S10H3-immunolabelled nuclei was significantly higher in the ipsilateral than in the contralateral side (ipsilateral: 72.33 ± 8.41 (n = 3), contralateral: 6.67 ± 3.48 (n = 3); p < 0.001, GLM; Fig. 6A). In contrast, no up-regulation of *p*-S10H3 expression was observed when only myelinated Aβ and Aδ primary afferent fibres were activated with low frequency stimuli (0.1 Hz; ipsilateral: 2.33 ± 1.20 (n = 3), contralateral: 1.67 ± 0.88; p = 0.948, GLM; Supplementary Figure 4B and Fig. 6A).

### Up-regulation of *p*-S10H3 expression in SSDHN depends on N-methyl-D-aspartate (NMDA) receptor activation

Up-regulation of *p*-ERK1/2 expression depends on the activation of the NMDA type glutamate receptors in spinal cord neurons^30^. To see whether S10H3 phosphorylation also requires NMDA receptor activation, a single dorsal root (L4 or L5) of *in vitro* intact spinal cord preparations of 2–3 week old rats was stimulated with C-fibre strength in the absence or presence of the NMDA receptor antagonist (2 *R*)-amino-5-phosphonovaleric acid (D-APV). To confirm the efficacy of electrical stimulation, primary afferent fibre stimulation-evoked EPSCs were monitored in SSDHN (Supplementary Figure 5).

In the absence of D-APV, the number of *p*-S10H3-expressing nuclei in the ipsilateral spinal dorsal horn was significantly increased (ipsilateral: 56.75 ± 6.70 (n = 4), contralateral: 5.75 ± 1.80 (n = 4), p < 0.001, GLM; Fig. 6B). D-APV significantly reduced this up-regulation (control: 56.75 ± 6.70 (n = 4), d-APV: 10.00 ± 1.96 (n = 4), p < 0.001, GLM; Fig. 6B).

### In addition to the NMDA receptor activity, up-regulation of *p*-S10H3 expression also depends on ERK1/2 and MSK1/2 activity in SSDHN

**Figure 7:**
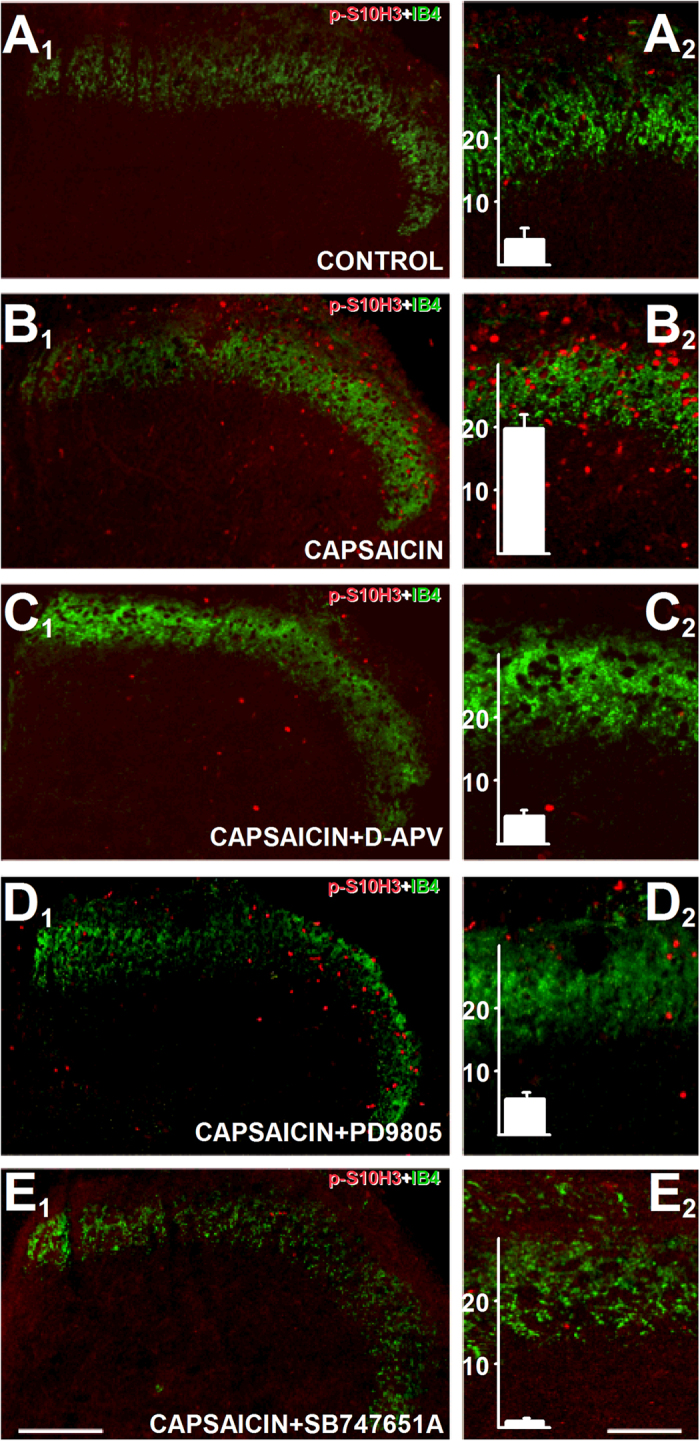
Phosphorylation of S10H3 by capsaicin involves the activation of the NMDA receptor, ERK1/2 and MSK1/2. (**A**_**1**_**–A**_**2**_)–(**E**_**1**_**–E**_**2**_) Images of sections cut from slices prepared from the L4-L5 spinal segments and exposed to various drugs followed by immunostaining with an anti-*p*-S10H3 antibody. *p*-S10H3-immunolabelled nuclei in spinal cord slice preparations exposed to vehicle (A_1_ and A_2_), 10 μM capsaicin (**B**_**1**_ and **B**_**2**_), 50 μM D-APV (NMDA receptor blocker) + 10 μM capsaicin (C_1_ and C_2_), 50 μM PD98052 (MEK1/2 inhibitor) + 10 μM capsaicin (**D**_**1**_ and **D**_**2**_) or 10 μM SB 747651 A (MSK1/2 inhibitor + 10 μM capsaicin (**E**_**1**_ and **E**_**2**_). IB4 labelling was done to label the ventral border of lamina II_i_. Chart inserts in (**A**_**2**_–**E**_**2**_) show results of quantification (n = 9). (Scale bars on (A_1_-E_1_), 200 μm; on (**A**_**2**_–**E**_**2**_), 100 μm.)

We studied the signalling mechanisms involved in the phosphorylation of S10H3 further in *in vitro* spinal cord slice preparations. Capsaicin application induced a significant up-regulation of *p*-S10H3 expression when capsaicin- and vehicle-exposed slices were compared (vehicle: 4.1 ± 1.5 (n = 9), capsaicin: 19.8 ± 1.9 (n = 9); p < 0.001, ANOVA followed by Dunnett post-hoc test; Fig. 7A1–2–B_1–2_). The majority of the *p*-S10H3-immunolabelled nuclei were found in the superficial laminae of the spinal cord, the ventral border of which was demonstrated by histochemical localisation of the binding sites for lectin *Bandeiraea simplicifolia* (IB4^5^; Fig. 7B1–2). Blocking NMDA receptor activity (D-APV; 50 μM) reduced the number of *p*-S10H3-immunopositive SSDHN to 4.4 ± 0.6 (n = 9; p < 0.0001; ANOVA followed by Dunnett test post-hoc; Fig. 7B1–2–C_1–2_). Blocking ERK1/2 activation by the MAPK inhibitor PD98052 (50 μM) also significantly reduced the number of SSDHN expressing *p*-S10H3 to 5.3 ± 0.7 (n = 9; p < 0.0001; ANOVA followed by Dunnett post-hoc test; Fig. 7B1–2 and D_1–2_). Finally, blocking the activity of MSK1/2 by SB747651A (10 μM)^31^, significantly reduced the up-regulation of *p*-S10H3 expression induced by capsaicin to 0.76 ± 0.28 (n = 9); p < 0.001, ANOVA followed by Dunnett post-hoc test; Fig. 7B1–2 and E_1–2_).

### Deleting MSK1/2 blocks S10H3 phosphorylation and c-Fos expression in SSDHN as well as the development of inflammatory heat hypersensitivity

**Figure 8:**
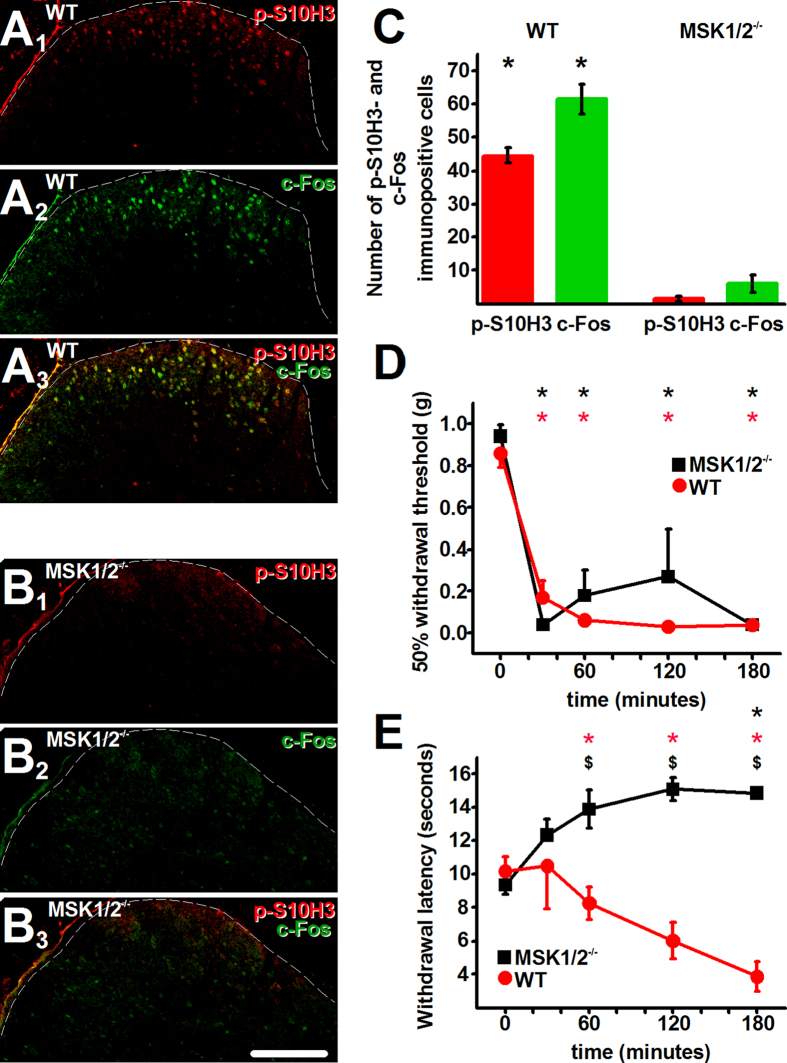
Carrageenan injection fails to up-regulate *p*-S10H3 and c-Fos expression and to induce heat hyperalgesia in MSK1/2^−/−^ mice. (**A**_**1**_–**A**_**3**_) An image of the ipsilateral side of a section cut from the L4-L5 spinal segments. *p*-S10H3- and c-Fos-expressing nuclei in the L4-L5 spinal cord of WT mice 3 hours after intraplantar carrageenan injection. (**B**_**1**_–**B**_**3**_) An image of the ipsilateral side of a section cut from the L4-L5 spinal segments. *p*-S10H3- and c-Fos-expressing nuclei in the L4-L5 spinal cord of MSK1/2^−/−^ mice 3 hours after intraplantar carrageenan injection. (Scale bar, 100 μm). (**C**) Quantification of *p*-S10H3- and c-Fos-immunopositive cells in WT mice and MSK1/2^−/−^ mice 3 hours after carrageenan injection. *p < 0.001, n = 4. (**D**) 50% mechanical withdrawal threshold for WT and MSK1/2^−/−^ mice after carrageenan injection. *p < 0.001 from baseline value, n = 4. (**E**) Withdrawal latency of WT and MSK1/2^−/−^ mice following injection carrageenan into the paw. *p < 0.003 from baseline value, ^$^p < 0.001 between WT and MSK1/2^−/−^, n = 4. Dashed lines indicate the white-grey matter border on each image. (Scale bar, 100 μm).

Carrageenan injection induces a prolonged inflammatory response and pain^32^. Inflammatory reactions (swelling and redness) appeared similar in WT and MSK1/2^−/−^ mice (data not shown). However, three hours after the injection, carrageenan induced up-regulation in *p*-S10H3 and c-Fos expression in the ipsilateral superficial spinal cord of WT but not of MSK1/2^−/−^ mice (*p*-S10H3: WT: 44.5 ± 2.33 (n = 4), MSK1/2^−/−^: 1.25 ± 0.75 (n = 4), GLM, p < 0.001; c-Fos: WT: 61.5 ± 4.37 (n = 4), MSK1/2^−/−^: 5.75 ± 2.75 (n = 4), GLM, p < 0.001; Fig. 8A1–3, B_1–3_ and C). Importantly, 83.21 ± 3.79% (n = 4) of the nuclei exhibiting *p*-S10H3-immunopositivity were also immunopositive for c-Fos, whereas 78.21 ± 3.13% (n = 4) of the nuclei immunopositive for c-Fos exhibited *p*-S10H3-immunolabelling in the ipsilateral spinal dorsal horn of WT animals. Saline injection did not induce up-regulation in either *p*-S10H3 or c-Fos expression in any part of the spinal cord (data not shown).

The carrageenan injection-evoked inflammation was accompanied by a rapid reduction of the 50% paw withdrawal threshold which indicated the development of mechanical allodynia both in WT and MSK1/2^−/−^ mice (Fig. 8D) and responses to mechanical stimuli were not significantly different in the two genotypes at any time point (Fig. 8D).

WT mice also exhibited a gradual reduction in the withdrawal latency to heat stimuli, which evidenced the development of heat hyperalgesia (Fig. 8E). In contrast, MSK1/2^−/−^ mice did not exhibit such reduction; instead they produced a gradual increase in the withdrawal latency, which reached the level of significance at 180 minutes (p = 0.003, n = 4, multiple ANOVA followed by Tukey’s post-hoc test; Fig. 8E). Due to these changes together, responses to noxious heat stimulation were significantly different at all time points between WT and MSK1/2^−/−^ mice (Fig. 8E).

## Discussion

Here we report the first PTM in a histone isoform, *p*-S10H3 which specifically occurs in the rat superficial spinal dorsal horn following the exposure of the hind limb to noxious heat that induces tissue injury and subsequent inflammation^20^, or capsaicin that following acute activation and rapid desensitisation also results in an inflammatory reaction^25^. In addition, repetitive electrical stimulation of nociceptive C fibres in rats or carrageenan injection into the mouse paw that evokes a delayed inflammatory reaction^32^ also up-regulate *p*-S10H3 expression in the superficial spinal dorsal horn. Capsaicin exposure and sustained repetitive electrical stimulation of rat nociceptive primary sensory neurons have similar effects *in vitro*. In contrast, low frequency electrical stimulation of myelinated primary sensory neurons or brief, non-tissue-damaging nociceptive stimuli fail to up-regulate *p*-S10H3 expression in rats. Importantly, the up-regulation in *p*-S10H3 expression significantly outlasts both the heat exposure and the acute excitation by capsaicin injection. Further, up-regulation in *p*-S10H3 expression is also evident 3 hours after carrageenan injection, when the inflammation is fully established and inflammatory mediators constitute the source for nociceptive activation. Together these data indicate that in addition to transient tissue-damaging, and inflammation-inducing stimuli, sustained stimuli by inflammatory mediators are also able to up-regulate *p*-S10H3 expression in the spinal dorsal horn.

Our co-expression studies indicate that only neurons exhibit *p*-S10H3 up-regulation in the superficial spinal dorsal horn. This finding is consistent with recent data that S10H3 phosphorylation occurs only in neurons in the nervous system^33^.

SSDHN are distributed in the area of the spinal cord, where nociceptive primary sensory neurons project and terminate^5^. Further, neurons with *p*-S10H3-expressing nuclei are often seen in close apposition with nociceptive primary sensory neuron terminals. Therefore, it is reasonable to propose that, at least, a proportion of neurons, which exhibit *p*-S10H3-immunopositive nuclei following prolonged nociceptive stimulation are second order neurons.

Capsaicin application, electrical stimulation, exposure to noxious heat and inflammatory mediators all induce the release of transmitters including glutamate that, following post-translational changes in second order neurons, are able to activate NMDA receptors expressed by those cells^2^,^3^,^6^,^34^. We found that blocking NMDA receptors significantly reduces both the capsaicin-induced and the persistent high frequency electrical stimulation-evoked phosphorylation of S10H3. Therefore, we also propose that NMDA receptor-mediated excitation of SSDNH induces S10H3 phosphorylation in the spinal dorsal horn.

NMDA receptor activation, among other effects, also activates ERK1/2 in SSDHN, and *p*-ERK1/2 is an established marker for spinal nociceptive processing^2^,^30^. In turn, *p*-ERK1/2 has been implicated in S10H3 phosphorylation through MSK1/2 activation^14^,^35^,^36^. Consistently, we found that blocking ERK1/2 activation blocks the capsaicin-induced up-regulation in *p*-S10H3 expression. Further, five minutes after burn injury, the great majority of *p*-ERK1/2-expressing SSDHN also expresses *p*-S10H3. While these findings support the role of *p*-ERK1/2 in S10H3 phosphorylation, only about 1/2–2/3 of *p*-S10H3-expressing cells express *p*-ERK1/2. This discrepancy could be due to rapid de-phosphorylation of *p*-ERK1/2, which is in an apparent contrast to the prolonged phosphorylation of S10H3. Alternatively, in addition to the ERK1/2 – MSK1/2-mediated pathway, another signalling cascade may also be involved in the phosphorylation of S10H3 in burn injury. Phosphorylated p38 (*p*-p38), another mitogen-activated protein kinase also activates MSK1/2^35^. However, although few neurons also exhibit *p*-p38 expression, the overwhelming majority of cells showing up-regulation in *p*-p38 expression are microglia in the spinal cord following peripheral inflammation^37^.

In spite of the functional link between ERK1/2 activation and S10H3 phosphorylation, up-regulation of *p*-ERK1/2 and *p*-S10H3 by noxious stimuli exhibits important further differences. First, the up-regulation of *p*-S10H3 expression lasts for a significantly longer time than up-regulation of *p-*ERK1/2 expression after capsaicin injection, which reflects differences in the kinetics of ERK1/2 and S10H3 phosphorylation. Second, while stimuli which produce tissue injury and/or inflammation and inflammatory conditions themselves induce phosphorylation both in S10H3 and ERK1/2, repeated brief application of nociceptive heat or pressure stimuli, which do not induce tissue damage and/or inflammation, is able to activate only ERK1/2. This latter difference suggests that brief non-tissue-damaging nociceptive stimuli and sustained tissue-damaging nociceptive stimuli respectively may activate ERK1/2 in different populations of cells.

In addition to *p*-ERK1/2, c-Fos is also an established marker for nociceptive processing in spinal cord neurons^17^. S10H3 phosphorylation leads to *fos* transcription^38^. Consistently, deleting MSK1/2 essentially blocks the expression of both *p*-S10H3 and c-Fos in SSDHN following carrageenan injection. Therefore, the small percentage of neurons which express either *p*-S10H3 or c-Fos alone in burn injury and carrageenan injection, could be due to the progression of the inflammatory reaction coupled with changes in activated/sensitised neurons in the spinal cord.

Deleting MSK1/2, in addition to preventing phosphorylation of S10H3, also prevents the development of heat hypersensitivity without having any effects on the development of mechanical allodynia following carrageenan injection. Correlations between the neurochemical properties and responsiveness of noxious stimuli of spinal second order neurons have been identified recently^5^,^39^,^40^,^41^, Hence, if the lack of heat hyperalgesia in carrageenan-injected MSK1/2^−/−^ mice is exclusively due to the lack of MSK1/2-evoked effects in spinal cord neurons (*vide infra*), *p*-S10H3 expression is up-regulated in neurons which respond only to noxious heat. Therefore, we regard *p*-S10H3 as a novel marker for the activation of SSDHN involved in the development of inflammatory heat hyperalgesia. Based on these findings, we also expect the presence of at least another sub-population of SSDNH, which is involved in the development of mechanical hypersensitivity, activated through a different pathway. Blocking ERK1/2 activation alleviates both inflammatory heat and mechanical hypersensitivity at least in certain inflammatory models^42^,^43^. Therefore, neurons, which express *p*-ERK1/2 but not *p*-S10H3 following the induction of burn injury may identify cells involved in the development of mechanical hypersensitivity. Nevertheless, our present findings suggest that neurons exhibiting *p*-S10H3 expression in inflammation of peripheral tissues are mainly, if not exclusively, excitatory cells, as *p*-S10H3 exhibited partial co-expression with the excitatory neuron marker calbindin, whereas no co-expression with the inhibitory neuron marker parvalbunin was found.

The development of hypoalgesia in naive or inflamed conditions has been reported previously and linked to various inhibitory mechanisms^44^. The presence of heat hypoalgesia in MSK1/2^−/−^ mice 3 hours after carrageenan injection hence suggests that, in peripheral inflammatory conditions, heat hypersensitivity does not, whereas inhibitory mechanisms do, develop in the absence of MSK1/2. These findings further indicate that antinociceptive mechanisms are also modality specific.

MSK1/2 are near ubiquitous, and through activating the cAMP response element-binding protein (CREB) and the RelA subunit of nuclear factor κ-B (NF-κB), and phosphorylating H3, are involved in various cellular processes^35^. At the periphery, these processes include the transformation, growth and spread of various cancer cells^45^ as well as regulating the innate immune response through up-regulating the expression of various molecules such as interleukin 10 or cyclooxygenase 2^46^,^47^. In the context of inflammation, loss of MSK1/2 activity results in increased sensitivity to endotoxic shock and allergic contact dermatitis suggesting that the roles of MSKs are largely anti-inflammatory^46^,^48^. In the brain, MSK1/2, through *p*-S10H3, contributes to learning and memory formation^10^,^11^,^35^,^38^. Hence, although the lack of heat hyperalgesia following carrageenan injection in MSK1/2^−/−^ mice may suggest that these kinases could be considered as targets for reducing pain in inflammation, this approach would require caution and could necessitate cell-specific targeting.

*p*-S10H3 activates the transcription of immediate early genes (IEG) including members of the *jun* and *fos* families, the early response gene 1, the activity-regulated cytoskeleton-associated protein gene (*arc*) and prostaglandin synthase 2 (*ptgs2*)^15^,^49^,^50^. Through that gene activating effect, phosphorylation of S10H3 results in transcriptional changes, and *p*-S10H3 expression exhibits positive correlation with IEG expression in the hippocampus^9^,^10^,^11^,^12^. The concomitant lack of up-regulation in *p*-S10H3 and c-Fos expression in the spinal cord in MSK1/2^−/−^ mice indicates that such correlation also exists in SSDHN.

Protein products of some *p*-S10H3-regulated IEG such as *arc* and *ptgs2* significantly contribute to sensitisation of neurons^16^,^51^. Protein products of other IEG regulate transcription of secondary response genes (SRG). In this context, it must be noted that through activation of SRG, c-Fos regulates neuronal activity in the hippocampus^52^. Importantly, protein products of many SRG, which are under the control of *p*-S10H30-regulated IEG, play a pivotal role in spinal nociceptive processing^16^,^18^,^53^.

In addition to being associated with IEG, p-S10H3 is also associated with histone cross-talk whereby S10H3 phosphorylation leads to modification of PTMs at other residues; namely *p*-S10H3 induces K14H3 acetylation and inhibits of K9H3 methylation and acetylation which both regulate gene transcription^54^. Intrathecal application of histone deacetylase inhibitors results in altered gene expression and reduced pain-related behaviour in various peripheral pathological conditions^55^,^56^. Hence, p-S10H3, in spinal cord neurons, may regulate transcriptional changes via multiple pathways.

In the present study, we assessed phosphorylation of S10H3 by noxious stimuli in SSDHN only. However, in addition to SSDHN, other neurons along the pain processing pathways may also respond to nociceptive signalling with S10H3 phosphorylation and subsequent activation of gene transcription. Further, the other MSK1/2 targets, CREB and NF-κB are involved, through regulating the transcription of nociception-related genes, in the development of inflammatory heat hyperalgesia and mechanical allodynia^2^,^57^. Nevertheless, we present convincing evidence that tissue injury/inflammation induces up-regulation of *p*-S10H3 expression in SSDHN. Although, with current technologies we cannot provide direct evidence, the data we present indicate that *p*-S10H3 is involved, in the spinal cord, in the regulation of genes, which contribute to sensitisation and the development of pain in inflammatory conditions. Therefore, up-regulation of *p*-S10H3 expression in SSDHN in peripheral inflammatory conditions may represent a starting point for the identification of genes, which, through their increased expression contribute to the development and maintenance of prolonged pain.

## Material and Methods

### Animals

All experiments were performed in accordance with the UK Animals (Scientific Procedures) Act 1986; Guidelines of the revised National Institutes of Health *Guide for the Care and Use of Laboratory Animals*, Directive 2010/63/EU of the European Parliament and of the Council on the Protection of Animals Used for Scientific Purposes and the Committee for Research and Ethical Issues of IASP published in Pain, 16 (1983) 109–110. Further, we obeyed to Good Laboratory Practice and ARRIVE guidelines. All procedures on animals were approved by veterinary services (Central Biological Services) at Imperial College London, UK, and Committees of Animal Research at the University of Debrecen, Hungary, and University of Szeged, Hungary. We took every effort to minimize the number and possible distress of animals. All animals were housed in climate-controlled rooms, on a 12 h light/dark cycle and with food and water *ad libitum*.

Altogether, 56 male Sprague-Dawley (150–200 g), 5 male Wistar rats (300–350 g) and 25 male Wistar rat pups (P14-28), 14 WT (C57BL6) and 14 MSK1/2^−/−^ mice (on C57BL6 background)^58^ were used. While Sprague-Dawley rats were used for *in vivo* experiments, Wistar rat were used to obtain *in vitro* spinal cord slice preparations. Wistar pups were used for *in vitro* dorsal root stimulation. WT and MSK1/2^−/−^ mice were used for *in vivo* studies. In our preliminary study we found no difference in the up-regulation of *p*-S10H3 expression in the spinal cord of Sprague-Dawley and Wistar rats (data are not shown).

### Drugs and reagents

Sodium pentobarbital was purchased from Euthatal (UK), urethane, carrageenan, PD98052 and gallamine triethiodide were purchased from Sigma (UK). Capsaicin, D-APV and SB747651A were purchased from Tocris (UK). Capsaicin was dissolved in ethanol (6%), Tween 80 (8%) and saline (86%). Carrageenan, urethane, D-APV and SB747651A were dissolved in saline. Due to regulations, different anaesthetics were used in different animal models of prolonged pain.

### Capsaicin injection

Sodium pentobarbital-anaesthetised (0.06 mg/g intraperitoneal) animals received capsaicin (25 μl of 3 mg/ml) or saline injection into one of the hind paws. At 5 minutes or 30 minutes survival times rats were terminally anaesthetised by a further dose of sodium pentobarbital and either perfused transcadially as described below or decapitated and their spinal cords removed for Western-blotting. Naive animals were used as negative controls.

### Noxious thermal stimulation

One of the hind paws of sodium pentobarbital-anaesthetised animals was dipped either into 4 °C or 44 °C water 6 times for 10 seconds each with 10 seconds intervals between each dipping. Five minutes after the start of the noxious stimulation, rats were terminally anaesthetised by a further injection of sodium pentobarbital and transcardially perfused as described below.

### Noxious mechanical stimulation

One of the hind paws of urethane-anaesthetised (0.02 mg/g intraperitoneal) rats were exposed to dorso-plantar compression with a pressure of 60 N/cm^2^ six times for 10 seconds each with 10 seconds released intervals between each compression. Rats were then terminally anaesthetised by further injection of urethane, and five minutes after the start of the noxious stimulation transcardially perfused as described below.

### Burn injury

We used the animal model of scalding type burn injury that we have established and described previously^21^. Briefly, under non-recoverable urethane anaesthesia (0.02 mg/g intraperitoneal) one of the hind paws up to the knee was immersed into 60 °C water for 2 minutes. Animals were then terminally anaesthetised with intraperitoneal sodium pentobarbital 5 minutes, 30 minutes or 1 hour after the burn injury. Fluctuations in anaesthesia level were checked through monitoring of the respiratory rate, corneal reflex and tail reflex and further urethane was injected if required. Naive animals and sham-injured animals (hind paw immersed into 37 °C for 2 minutes) were used as controls.

### Electrical stimulation

Electrical stimulation was performed both in *in vivo* and *in vitro. In vivo*, the trachea, femoral vein and femoral nerve of sodium pentobarbital-anaesthetised animals were exposed. The trachea was cannulated and rats were ventilated. The nerve was placed onto a bipolar platinum wire electrode, whereas the vein was cannulated for injecting 5–10 mg/ml of gallamine triethiodide in order to inhibit muscle contractions during electrical stimulation which was performed by delivering ten trains of two hundreds pulses of 30 V amplitude and 2ms width at 10 Hz frequency, or ten trains of two pulses of 15 V amplitude and 100 μs width with 0.1 Hz frequency in every 30 seconds for 5 minutes. Rats were then terminally anaesthetised by sodium pentobarbital and processed as described below.

For *in vitro* experiments, animals were terminally anaesthetised with sodium pentobarbital and decapitated. The vertebral column was quickly removed and immersed in oxygenated artificial cerebrospinal fluid (in mM: NaCl, 120; KCl, 2.5; NaHCO3, 26; glucose, 10; NaH2PO4, 1.25; CaCl2, 2; MgCl2, 1; myo-inositol, 3; ascorbic acid, 0.5; and sodium-pyruvate, 2; pH 7.2) at room temperature. The lumbar spinal cord with unilateral L4 or L5 dorsal roots attached was dissected. The spinal cord was glued to a golden plate with cyanoacrylate adhesive and transferred to the recording chamber. The dorsal roots, which had a length of 7–11 mm, were stimulated via a suction electrode fabricated from a borosilicate glass capillary and a BioStim STC-7a stimulator (Supertech, Hungary). Electrical pulses of 500 μA in amplitude and 1 ms in width were delivered at 2 Hz for 15 seconds, followed by 15 seconds without stimulation. This protocol was repeated four times and the preparation was left for a 3-minute recovery before fixation in 4% paraformaldehyde. All stimulations and measurements were done at 22–24 °C.

The effectiveness of dorsal root electrical stimulation was tested by monitoring primary afferent activity-evoked currents of superficial dorsal horn neurons. Whole-cell patch-clamp recordings were performed at room temperature using an EPC 10 Double amplifier (HEKA, Germany). Patch pipettes with 6–8 MΩ pipette resistance were filled with a solution containing (in mM): K-gluconate, 120; NaCl, 5; 4-(2-hydroxyethyl)-1- piperazineethanesulfonic acid (HEPES), 10; EGTA, 2; CaCl2, 0.1; Mg-ATP, 5; Na3-GTP, 0.3; Na2- phosphocreatinine, 10; biocytin, 8; pH 7.3.

### *In vitro* pharmacology

Following terminal anaesthesia with sodium pentobarbital and decapitation, the lumbar vertebral column was exposed through a midline skin incision and removed by transecting it at about the 1^st^ lumbar and 6^th^ lumbar vertebral levels. The spinal cord was ejected by injecting 5–10 ml of artificial cerebrospinal fluid (ACSF, composition in mM: 119 NaCl, 26.2 NaHCO_3_, 2.5 KCl, 1 NaH_2_PO_4_, 1.3 MgCl_2_, 2.5 CaCl_2_ and 10 glucose, pH = 7.4, saturated with 95% O_2_ and 5% CO_2_) through the caudal end of the spinal canal. Then, about 1.5 mm thick slices of the lumbar spinal cord were cut with a razor blade and transferred into carbogen-gassed ACSF medium maintained at 37 °C. After a resting period of 30 minutes, slices were exposed to capsaicin (10 μM) or one of the following antagonists: D-APV (50 μM), PD98052 (50 μM) or SB-747651A (10 μM) for 20 minutes. Antagonist exposure was followed by exposure of the slices to saline or the same antagonist together with capsaicin (10 μM) for 20 min. For an additional control, the vehicle (6% ethanol, 8% Tween 80 in saline) for capsaicin was applied. A respresentative image of immunostaining after the incubation of slices in SB-747651A (10 μM) followed by saline is shown in Supplementary Figure 6. Three hours later, tissue slices were fixed with a buffered solution of 4% paraformaldehyde for 3 h at 4 °C, and then transferred and stored in a phosphate buffer solution containing sodium azide (0.1%) until further processing. Prior to sectioning on a cryostat, tissue specimens were kept in a buffered sucrose (30%) solution for 12–24 h. Free-floating sections 15 μm in thickness were then processed for immunolabelling.

### Harvesting tissues

After inducing terminal anaesthesia with sodium pentobarbital, animals were perfused transcardially with saline containing 0.5% heparin (Leo Pharma) followed either by 4% paraformaldehyde (Acros Organic) dissolved in 0.01 M phosphate-buffered saline (PBS) or 1% paraformaldehyde in 0.1 M phosphate buffer supplemented with 0.5% methanol. The L2-L6 spinal cord was carefully dissected, post-fixed the same fixative for overnight.

### Western-blotting

After burn injury, the L4-L5 spinal segments were collected and hemisected. Proteins were extracted with the EpiQuick Total Histone Extraction Kit (Epigentek, USA) according to the manufacturer’s instructions. Following capsaicin injection, ipsilateral and contralateral cords from two animals were pooled, respectively, to increase the amount of phosphorylated histones within the sample. Samples were then processed for histone extraction.

Samples were denatured at 95 °C for 5 minutes and loaded onto NuPAGE Novex 12% Bis-Tris Protein Gels (Invitrogen, UK). Samples were then dry transferred onto PVDF membranes (Invitrogen, UK). Membranes were incubated with 5% non-fat milk powder (Sigma, UK) for 1 hour at room temperature then incubated with an anti-p-S10H3 and β-actin antibodies at 4 °C for an overnight (Supplementary Table 1). Secondary antibodies were applied at room temperature for 1 hour and the reaction was visualised with the Luminol kit (Santa Cruz, USA; Supplementary Tables 2). Membranes were examined in a G:Box (SynGene, UK) using the GeneSnap software package (Synoptics LTd, SynGene version 7.12.06). Membranes were then stripped with Restore Western Blot Stripping Buffer (Thermo Scientific, UK) and reacted again with an anti-pan-H3 antibody. For control, another set of Western blots was run with the same samples and conditions but in reverse order of reactions. No differences in results were observed. Non-immune IgG controls were also performed when no proteins were identified (Supplementary Figure 7). The expected molecular weight for *p*-S10H3 and β-actin are 17 kD and 42 kD, respectively.

### Immunolabelling

Fixed tissue samples were immersed into 30% sucrose in PBS until they sank. Tissue samples were then embedded in Tissue-Teck OCT compound (VWR, UK) and 10 μm transverse sections of the L4-L5 segments of the spinal cords were cut on a cryostat. Sections were then thaw-mounted on Superfrost Plus microscope slides (Thermo Scientific, UK). Every tenth section was put on the same slide hence adjacent sections were separated by 100 μm on each slide.

Slides were incubated in PBS containing 0.3% Triton-X 100 (PBST) for 10 minutes, then incubated in 10% normal donkey serum (NDS) for 1 hour. Sections were then incubated in the primary antibody (Supplementary Table 1) at 4 °C for overnight in a humidified chamber. In the majority of sections, Alexa Fluor-conjugated secondary antibodies were used for visualisation (Supplementary Table 2). In some sections visualisation was done by applying biotin-conjugated secondary antibody followed by streptavidin-conjugated horseradish peroxidase and the nickel diamino-benzidine reaction using the ABC kit (Vector Labs, UK). Slides were coverslipped with Vectashield mounting medium (Vector Laboratories, UK), home-made mowiol medium or ProlongGold antifade medium (ThermoFisher Scientific, Carlsbad, CA, USA). Sections used for double or triple staining, were processed either in consecutive or simultaneous reactions.

The tyramide signal amplification procedure was used when the host species of two primary antibodies used in combinations were the same. Here, the endogenous peroxidase activity was quenched by incubating the sections in 70% methanol with 0.3% of H_2_O_2_, whereas unspecific binding of biotin was blocked with the Avidin/Biotin blocking kit (Vector, UK). Following the incubation of sections in the primary antibody, slides were washed with PBST, incubated for one hour in a biotinylated secondary antibody in 2% NDS in PBST, then in the ABC complex (1:200; Vector Labs) in PBST for an hour. This was followed by incubation with biotinyl tyramide (1:50; Perkin-Elmer Inc., UK) for 5 minutes. Subsequently, slides were incubated with Alexa Fluor Streptavidin in PBST for an hour. The second reaction was then performed as described above. Subsequent steps in all immunolabelling protocol were separated by a 5 minutes wash in PBST. All the washes were performed at room temperature in PBST, with the addition of 2% NDS for the dilution of the antibodies. In some experiments we also performed antigen retrieval which included incubating sections in sodium citrate buffer (10 mM; pH 6.0) containing 0.05% Tween-20 at 90 °C for 5 min. No apparent differences were found between results obtained using the various protocols.

Controls for all immmunolabelling were performed by replacing the primary antiserum with normal donkey serum. For the TSA reaction, unamplified slides and amplified negative controls were prepared as controls. No immunostaining was observed in control experiments. Result of such control for the anti-*p*-S10H3 antibody is shown in Supplementary Figure 8.

Cryostat sections cut from *in vitro* spinal cord slice preparations were also reacted for the demonstration of *p*-S10H3 and isolectin IB4-binding using the indirect immunofluorescence technique and lectin histochemistry. *p*-S10H3-immunolabeling was visualized with cy3-labelled polyclonal donkey anti-rabbit IgG. Isolectin B4 binding was demonstrated with biotin-conjugated isolectin B4 (IB4) from *Bandeiraea simplicifolia* (L2140, 1:1000, Sigma, USA) using a FITC-avidin conjugate (1:500, Jackson ImmunoResearch Laboratories, West Grove, PA, USA).

### Behavioural assessment

Following determining baseline responses to heat and mechanical stimuli (see below), animals were anaesthetised briefly by isoflurane and injected either with saline (25 μl) or 1.5% carrageenan (25 μl) into one of their hind paws. Thirty minutes, 1 hour, 2 hours and 3 hours after the injection, animals were assessed for the development of heat hyperalgesia with a hot plate (WPI, UK) and mechanical allodynia with von Frey filaments (Bioseb, USA). Animals were trained for 1 hour on each of the two preceding days of the experiment.

On the day of the experiment, for the assessment of mechanical allodynia, mice were placed in a Perspex chamber with a 0.8 cm-diameter mesh flooring and allowed to acclimatize for 30 min. Calibrated von Frey hairs (0.008–2 g, Bioseb, USA) were applied to the plantar surface of the hind paw, until the fibre was bent, and left for 3 seconds or until animal withdrew the paw. A series of successive hairs were then applied, according to the up-down method. The 50% paw withdrawal value was then calculated.

For assessing heat hyperalgesia, mice were placed on a hot plate set to 52 °C and the time until they showed pain-related behaviour (shaking or licking the paw) was established. If an animal did not exhibit any pain-related behaviour within 20 second, the exposure to heat was stopped in order to prevent injury. The paw withdrawal latency was assessed 3 times for each animal at each time point. Care was taken not to repeat testing the same animal within 5 minutes.

### Data analysis and statistics

Immunostained sections were examined either with a Leica DMBL (Leica, Germany) fluorescence microscope equipped with a Retiga 2000 R digital camera (QImaging, USA) or with a Hamamatsu Colour Chilled 3CCD camera, or under a Zeiss LSM710 or an Olympus FV1000 confocal microscope.

Immunopositive nuclei from *in vivo* experiments and *in vitro* whole spinal cord preparations were counted both in the ipsilateral and contralateral sides of six sections found in a single slide. The sum of neurons counted in the six sections was used for analysis. The number of neurons both on the ipsilateral and contralateral side of animals, which had received the same treatment, was averaged and differences were compared. The comparison included using the generalised linear model (GLM), which is more flexible than the general linear model and can be used even if the variables do not meet criteria of homocedasticity^59^. For establishing the statistical significance of the differences the Wald chi square test followed by a pertinent pairwise comparison, if needed, was used.

Stereological methods were used to quantify p-S10H3-positive cells in the superficial layers (Rexed’s lamina I and II) of the spinal dorsal horn of sections from the *in vitro* spinal cord slice preparations. The ventral extension of the IB4-staining was defined as the ventral border of lamina II. The number of p-S10H3-positive cells was counted in randomly selected three areas of interest (AOI) of three sections obtained from each animal from 3 different experiments. The AOI was defined as an area of 3 × 10^4^ μm^2^ of the superficial laminae of the dorsal horn as defined above. Statistical comparison of data was done by ANOVA followed by the Dunnett post-hoc test.

For Western blot, the density ratios of the β-actin-normalised p-S10H3 densities of ipsi- and contralateral sides were calculated and compared with the Student’s *t*-test.

The statistical analysis of behavioural data was performed between withdrawal responses (at different testing times or between different animal groups at the same testing time) using multiple ANOVA followed by the Tukey’s test.

Data are expressed as mean + /−standard error mean, n refers to the number of biological repetitions. Differences were regarded significant at p < 0.05. p values are given as exact values above p = 0.001. Below that value p is give as p < 0.001.

## Additional Information

**How to cite this article**: Torres-Pérez, J. V. *et al*. Phosphorylated Histone 3 at Serine 10 Identifies Activated Spinal Neurons and Contributes to the Development of Tissue Injury-Associated Pain. *Sci. Rep.* **7**, 41221; doi: 10.1038/srep41221 (2017).

**Publisher's note:** Springer Nature remains neutral with regard to jurisdictional claims in published maps and institutional affiliations.

## Supplementary Material

Supplementary Information

Supplementary Dataset

## References

[b1] Breivik H., Collett B., Ventafridda V., Cohen R. & Gallacher D. Survey of chronic pain in Europe: prevalence, impact on daily life, and treatment. Eur. J. Pain 10, 287–333 (2006). 16095934 10.1016/j.ejpain.2005.06.009

[b2] Ji R. R., Kohno T., Moore K. A. & Woolf C. J. Central sensitization and LTP: do pain and memory share similar mechanisms? Trends Neurosci. 26, 696–705 (2003). 14624855 10.1016/j.tins.2003.09.017

[b3] Ganguly K. & Poo M. M. Activity-dependent neural plasticity from bench to bedside. Neuron 80, 729–741 (2013). 24183023 10.1016/j.neuron.2013.10.028

[b4] Edelmayer R. M., Brederson J. D., Jarvis M. F. & Bitner R. S. Biochemical and pharmacological assessment of MAP-kinase signaling along pain pathways in experimental rodent models: a potential tool for the discovery of novel antinociceptive therapeutics. Biochem. Pharmacol. 87, 390–398 (2014). 24300134 10.1016/j.bcp.2013.11.019

[b5] Todd A. J. Neuronal circuitry for pain processing in the dorsal horn. Nat. Rev. Neurosci. 11, 823–836 (2010). 21068766 10.1038/nrn2947 PMC3277941

[b6] Sandkuhler J. Models and mechanisms of hyperalgesia and allodynia. Physiol. Rev. 89, 707–758 (2009). 19342617 10.1152/physrev.00025.2008

[b7] Ng M. K. & Cheung P. A brief histone in time: understanding the combinatorial functions of histone PTMs in the nucleosome context. Biochem. Cell. Biol. 1–10 (2015). 10.1139/bcb-2015-0031 26197985

[b8] Kornberg R. D. Chromatin structure: a repeating unit of histones and DNA. Science 184, 868–871 (1974). 4825889 10.1126/science.184.4139.868

[b9] Chandramohan Y., Droste S. K., Arthur J. S. & Reul J. M. The forced swimming-induced behavioural immobility response involves histone H3 phospho-acetylation and c-Fos induction in dentate gyrus granule neurons via activation of the N-methyl-D-aspartate/extracellular signal-regulated kinase/mitogen- and stress-activated kinase signalling pathway. Eur. J. Neurosci. 27, 2701–2713 (2008). 18513320 10.1111/j.1460-9568.2008.06230.x

[b10] Chwang W. B., Arthur J. S., Schumacher A. & Sweatt J. D. The nuclear kinase mitogen- and stress-activated protein kinase 1 regulates hippocampal chromatin remodeling in memory formation. J. Neurosci. 27, 12732–12742 (2007). 18003853 10.1523/JNEUROSCI.2522-07.2007 PMC5724774

[b11] Correa S. A. . MSK1 regulates homeostatic and experience-dependent synaptic plasticity. J. Neurosci. 32, 13039–13051 (2012). 22993422 10.1523/JNEUROSCI.0930-12.2012 PMC6621478

[b12] Carter S. D., Mifsud K. R. & Reul J. M. Distinct epigenetic and gene expression changes in rat hippocampal neurons after Morris water maze training. Front. Behav. Neurosci. 9, 156 (2015). 26136669 10.3389/fnbeh.2015.00156 PMC4468857

[b13] Mahadevan L. C., Willis A. C. & Barratt M. J. Rapid histone H3 phosphorylation in response to growth factors, phorbol esters, okadaic acid, and protein synthesis inhibitors. Cell 65, 775–783 (1991). 2040014 10.1016/0092-8674(91)90385-c

[b14] Thomson S. . The nucleosomal response associated with immediate-early gene induction is mediated via alternative MAP kinase cascades: MSK1 as a potential histone H3/HMG-14 kinase. EMBO J. 18, 4779–4793 (1999). 10469656 10.1093/emboj/18.17.4779 PMC1171550

[b15] Nowak S. J. & Corces V. G. Phosphorylation of histone H3 correlates with transcriptionally active loci. Genes Dev. 14, 3003–3013 (2000). 11114889 10.1101/gad.848800 PMC317109

[b16] Keum Y. S., Kim H. G., Bode A. M., Surh Y. J. & Dong Z. UVB-induced COX-2 expression requires histone H3 phosphorylation at Ser10 and Ser28. Oncogene 32, 444–452 (2013). 22391560 10.1038/onc.2012.71 PMC3504182

[b17] Hunt S. P., Pini A. & Evan G. Induction of c-fos-like protein in spinal cord neurons following sensory stimulation. Nature 328, 632–634 (1987). 3112583 10.1038/328632a0

[b18] Naranjo J. R., Mellstrom B., Achaval M. & Sassone-Corsi P. Molecular pathways of pain: Fos/Jun-mediated activation of a noncanonical AP-1 site in the prodynorphin gene. Neuron 6, 607–617 (1991). 1901718 10.1016/0896-6273(91)90063-6

[b19] Tochiki K. K., Maiaru M., Norris C., Hunt S. P. & Geranton S. M. The mitogen and stress-activated protein kinase 1 regulates the rapid epigenetic tagging of dorsal horn neurons and nocifensive behaviour. Pain 157, 2594–2604 (2016). 27482631 10.1097/j.pain.0000000000000679 PMC5065054

[b20] Laycock H., Valente J., Bantel C. & Nagy I. Peripheral mechanisms of burn injury-associated pain. Eur. J. Pharmacol. 716, 169–178 (2013). 23500208 10.1016/j.ejphar.2013.01.071

[b21] White J. P. . Severe burn injury induces a characteristic activation of extracellular signal-regulated kinase 1/2 in spinal dorsal horn neurons. Eur. J. Pain 15, 683–690 (2011). 21371920 10.1016/j.ejpain.2010.12.006

[b22] Kapuscinski J. DAPI: a DNA-specific fluorescent probe. Biotech. Histochem. 70, 220–233 (1995). 8580206 10.3109/10520299509108199

[b23] Nagy I., Friston D., Valente J. S., Torres Perez J. V. & Andreou A. P. Pharmacology of the capsaicin receptor, transient receptor potential vanilloid type-1 ion channel. Prog. Drug Res. 68, 39–76 (2014). 24941664 10.1007/978-3-0348-0828-6_2

[b24] Boros K. . Multiple impairments of cutaneous nociceptor function induced by cardiotoxic doses of Adriamycin in the rat. Naunyn Schmiedebergs Arch. Pharmacol. 389, 1009–1020 (2016). 27342418 10.1007/s00210-016-1267-x

[b25] Jancso N., Jancso-Gabor A. & Szolcsanyi J. Direct evidence for neurogenic inflammation and its prevention by denervation and by pretreatment with capsaicin. Br. J. Pharmacol. Chemother. 31, 138–151 (1967). 6055248 10.1111/j.1476-5381.1967.tb01984.x PMC1557289

[b26] Caterina M. J. . The capsaicin receptor: a heat-activated ion channel in the pain pathway. Nature 389, 816–824 (1997). 9349813 10.1038/39807

[b27] Tominaga M. . The cloned capsaicin receptor integrates multiple pain-producing stimuli. Neuron 21, 531–543 (1998). 9768840 10.1016/s0896-6273(00)80564-4

[b28] Simone D. A., Baumann T. K. & LaMotte R. H. Dose-dependent pain and mechanical hyperalgesia in humans after intradermal injection of capsaicin. Pain 38, 99–107 (1989). 2780068 10.1016/0304-3959(89)90079-1

[b29] Hoffmann T., Sauer S. K., Horch R. E. & Reeh P. W. Sensory transduction in peripheral nerve axons elicits ectopic action potentials. J. Neurosci. 28, 6281–6284 (2008). 18550771 10.1523/JNEUROSCI.1627-08.2008 PMC6670532

[b30] Ji R. R., Baba H., Brenner G. J. & Woolf C. J. Nociceptive-specific activation of ERK in spinal neurons contributes to pain hypersensitivity. Nat. Neurosci. 2, 1114–1119 (1999). 10570489 10.1038/16040

[b31] Naqvi S. . Characterization of the cellular action of the MSK inhibitor SB-747651A. Biochem. J. 441, 347–357 (2012). 21970321 10.1042/BJ20110970

[b32] van Arman C. G., Carlson R. P., Risley E. A., Thomas R. H. & Nuss G. W. Inhibitory effects of indomethacin, aspirin and certain other drugs on inflammations induced in rat and dog by carrageenan, sodium urate and ellagic acid. J. Pharmacol. Exp. Ther. 175, 459–468 (1970). 5481710

[b33] Mackowiak M., Guzik R., Dudys D., Bator E. & Wedzony K. MK-801, a NMDA receptor antagonist, increases phosphorylation of histone H3 in the rat medial prefrontal cortex. Pharmacol. Rep. 65, 1112–1123 (2013). 24399707 10.1016/s1734-1140(13)71469-5

[b34] Woolf C. J. & Thompson S. W. The induction and maintenance of central sensitization is dependent on N-methyl-D-aspartic acid receptor activation; implications for the treatment of post-injury pain hypersensitivity states. Pain 44, 293–299 (1991). 1828878 10.1016/0304-3959(91)90100-C

[b35] Arthur J. S. MSK activation and physiological roles. Front. Biosci. 13, 5866–5879 (2008). 18508628 10.2741/3122

[b36] Soloaga A. . MSK2 and MSK1 mediate the mitogen- and stress-induced phosphorylation of histone H3 and HMG-14. EMBO J. 22, 2788–2797 (2003). 12773393 10.1093/emboj/cdg273 PMC156769

[b37] Hua X. Y. . Intrathecal minocycline attenuates peripheral inflammation-induced hyperalgesia by inhibiting p38 MAPK in spinal microglia. Eur. J. Neurosci. 22, 2431–2440 (2005). 16307586 10.1111/j.1460-9568.2005.04451.x

[b38] Gutierrez-Mecinas M. . Long-lasting behavioral responses to stress involve a direct interaction of glucocorticoid receptors with ERK1/2-MSK1-Elk-1 signaling. Proc. Natl. Acad. Sci. USA 108, 13806–13811 (2011). 21808001 10.1073/pnas.1104383108 PMC3158237

[b39] Uta D. . TRPA1-expressing primary afferents synapse with a morphologically identified subclass of substantia gelatinosa neurons in the adult rat spinal cord. Eur. J. Neurosci. 31, 1960–1973 (2010). 20497466 10.1111/j.1460-9568.2010.07255.x PMC4232817

[b40] Duan B. . Identification of spinal circuits transmitting and gating mechanical pain. Cell 159, 1417–1432 (2014). 25467445 10.1016/j.cell.2014.11.003 PMC4258511

[b41] Polgar E. . Functional differences between neurochemically defined populations of inhibitory interneurons in the rat spinal dorsal horn. Pain 154, 2606–2615 (2013). 23707280 10.1016/j.pain.2013.05.001 PMC3858808

[b42] van den Heuvel I., Reichl S., Segelcke D., Zahn P. K. & Pogatzki-Zahn E. M. Selective prevention of mechanical hyperalgesia after incision by spinal ERK1/2 inhibition. Eur. J. Pain. 19, 225–235 (2015). 24976579 10.1002/ejp.540

[b43] Ji R. R., Befort K., Brenner G. J. & Woolf C. J. ERK MAP kinase activation in superficial spinal cord neurons induces prodynorphin and NK-1 upregulation and contributes to persistent inflammatory pain hypersensitivity. J. Neurosci. 22, 478–485 (2002). 11784793 10.1523/JNEUROSCI.22-02-00478.2002 PMC6758654

[b44] Rezende R. M. . Different mechanisms underlie the analgesic actions of paracetamol and dipyrone in a rat model of inflammatory pain. Br. J. Pharmacol. 153, 760–768 (2008). 18157167 10.1038/sj.bjp.0707630 PMC2259218

[b45] Dunn K. L., Espino P. S., Drobic B., He S. & Davie J. R. The Ras-MAPK signal transduction pathway, cancer and chromatin remodeling. Biochem. Cell. Biol. 83, 1–14 (2005). 15746962 10.1139/o04-121

[b46] Ananieva O. . The kinases MSK1 and MSK2 act as negative regulators of Toll-like receptor signaling. Nat. Immunol. 9, 1028–1036 (2008). 18690222 10.1038/ni.1644

[b47] MacKenzie K. F. . MSK1 and MSK2 inhibit lipopolysaccharide-induced prostaglandin production via an interleukin-10 feedback loop. Mol. Cell. Biol. 33, 1456–1467 (2013). 23382072 10.1128/MCB.01690-12 PMC3624268

[b48] Bertelsen T. . The role of mitogen- and stress-activated protein kinase 1 and 2 in chronic skin inflammation in mice. Exp. Dermatol. 20, 140–145 (2011). 21166721 10.1111/j.1600-0625.2010.01153.x

[b49] Reul J. M., Hesketh S. A., Collins A. & Mecinas M. G. Epigenetic mechanisms in the dentate gyrus act as a molecular switch in hippocampus-associated memory formation. Epigenetics 4, 434–439 (2009). 19829071 10.4161/epi.4.7.9806

[b50] Chandramohan Y., Droste S. K. & Reul J. M. Novelty stress induces phospho-acetylation of histone H3 in rat dentate gyrus granule neurons through coincident signalling via the N-methyl-D-aspartate receptor and the glucocorticoid receptor: relevance for c-fos induction. J. Neurochem. 101, 815–828 (2007). 17250652 10.1111/j.1471-4159.2006.04396.x

[b51] Bramham C. R., Worley P. F., Moore M. J. & Guzowski J. F. The immediate early gene arc/arg3.1: regulation, mechanisms, and function. J. Neurosci. 28, 11760–11767 (2008). 19005037 10.1523/JNEUROSCI.3864-08.2008 PMC2615463

[b52] Zhang J. . c-fos regulates neuronal excitability and survival. Nat. Genet. 30, 416–420 (2002). 11925568 10.1038/ng859

[b53] Koh Y. H., Tamizhselvi R. & Bhatia M. Extracellular signal-regulated kinase 1/2 and c-Jun NH2-terminal kinase, through nuclear factor-kappaB and activator protein-1, contribute to caerulein-induced expression of substance P and neurokinin-1 receptors in pancreatic acinar cells. J. Pharmacol. Exp. Ther. 332, 940–948 (2010). 20007404 10.1124/jpet.109.160416

[b54] Latham J. A. & Dent S. Y. Cross-regulation of histone modifications. Nat. Struc. Mol. Biol. 14, 1017–1024 (2007). 10.1038/nsmb1307 17984964

[b55] Bai G., Wei D., Zou S., Ren K. & Dubner R. Inhibition of class II histone deacetylases in the spinal cord attenuates inflammatory hyperalgesia. Mol. Pain 6, 51 (2010). 20822541 10.1186/1744-8069-6-51 PMC2942827

[b56] Crow M. . HDAC4 is required for inflammation-associated thermal hypersensitivity. FASEB J. 29, 3370–3378 (2015). 25903105 10.1096/fj.14-264440 PMC4511203

[b57] Svensson C. I. & Yaksh T. L. The spinal phospholipase-cyclooxygenase-prostanoid cascade in nociceptive processing. Annu. Rev. Pharmacol. Toxicol. 42, 553–583 (2002). 11807183 10.1146/annurev.pharmtox.42.092401.143905

[b58] Wiggin G. R. . MSK1 and MSK2 are required for the mitogen- and stress-induced phosphorylation of CREB and ATF1 in fibroblasts. Mol. Cell Biol. 22, 2871–2881 (2002). 11909979 10.1128/MCB.22.8.2871-2881.2002 PMC133730

[b59] McCulloch C. E., Searle S. R. & Neuhaus J. M. Generalized, Linear, and Mixed Models, 2nd Edition. 2nd edn, (Wiley- InterScience, 2008).

