# Supplementary material for: Phosphorylated Histone 3 at Serine 10 Identifies Activated Spinal Neurons and Contributes to the Development of Tissue Injury-Associated Pain
Source: Sci Rep. 2017 Jan 25;7:41221. doi: 10.1038/srep41221 (PMC5264160; doi:10.1038/srep41221)
Supplement: Supplementary Information [file srep41221-s1.doc]

**SUPPLEMENTARY FIGURES**

**PHOSPHORYLATED HISTONE 3 AT SERINE 10 IDENTIFIES ACTIVATED SPINAL NEURONS AND CONTRIBUTES TO THE DEVELOPMENT OF TISSUE INJURY-ASSOCIATED PAIN**

Jose Vicente Torres-Pérez^1^, Péter Sántha^2^, Angelika Varga^3,4^, Peter Szucs^3,4^, Joao Sousa-Valente^1^; Botond Gaal^4^, Miklós Sivadó^3^, Anna P Andreou^1^, Sara Beattie^1^, Bence Nagy^5^, Klara Matesz^4^, J. Simon C. Arthur^6^, Gábor Jancsó^2^ and Istvan Nagy^1$^

^1^Nociception Group, Section of Anaesthetics, Pain Medicine and Intensive Care, Department of Surgery and Cancer, Imperial College London, London, SW10 9NH, United Kingdom; ^2^Department of Physiology, University of Szeged, Szeged, H-6720, Hungary; ^3^MTA-DE-NAP B-Pain Control Research Group, Department of Anatomy, Histology and Embryology and Department of Physiology, University of Debrecen, Debrecen, H-4012, Hungary; ^4^Department of Anatomy, Histology and Embryology, University of Debrecen, Debrecen, H-4012, Hungary; ^5^The Ipswich Hospital, Ipswich, IP4 5PD, United Kingdom; ^6^Division of Cell Signalling and Immunology, College of Life Sciences, Sir James Black Centre, University of Dundee, Dundee DD1 5EH, United Kingdom

Running title: p-S10H3 in spinal nociceptive processing

**Supplementary Figure 1**


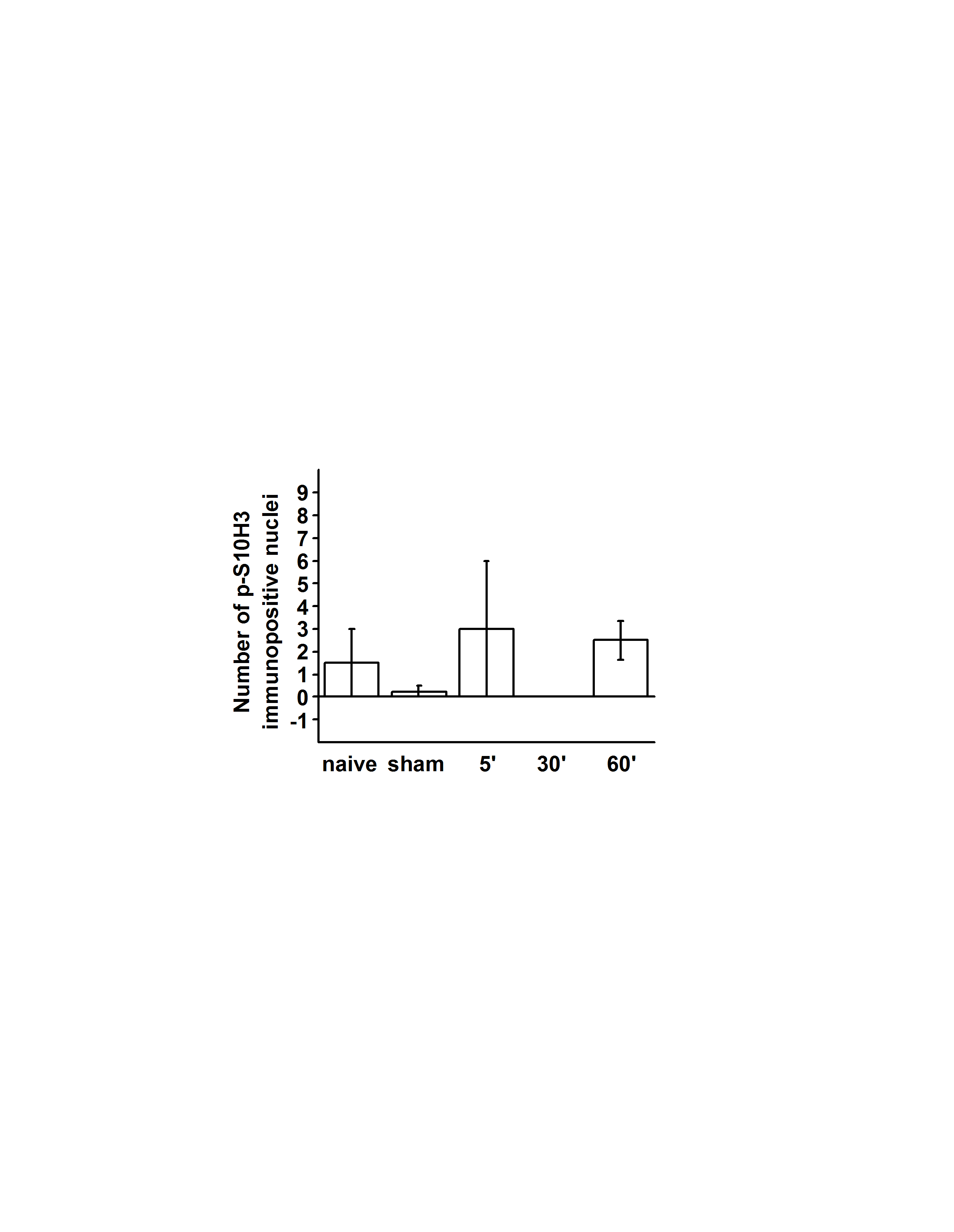


Number of p-S10H3 immunopositve nuclei in contralateral side of the superficial spinal dorsal horn of naive (naive), sham-injured (sham), and burn-injured rats 5 minutes (5’), 30 minutes (30’) and 60 minutes (60’) after the injury. Neither sham injury nor burn injury induces up-regulation of p-S10H3 expression in the contralateral spinal cord.

**Supplementary Figure 2**

**
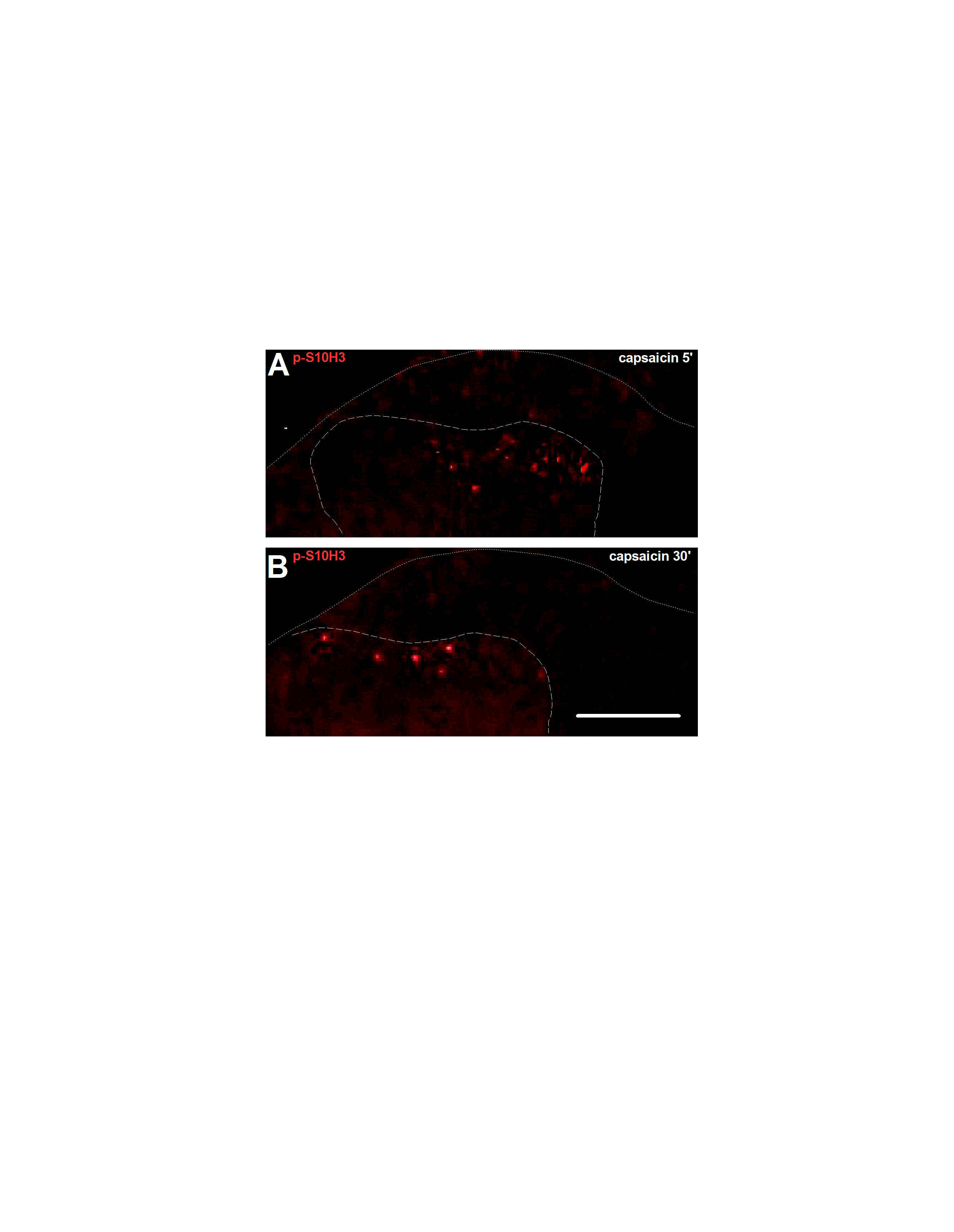
**

(A) An image of a section cut from the ipsilatreal side of the L4-L5 spinal segments 5 minutes after capsaicin injection into the paw. The injection resulted in the expression of *p*-S10H3 in a group of SSDHN.

(B) An image of a section cut from the ipsilatreal side of the L4-L5 spinal segments 30 minutes after injectiong capsaicin into the paw. *p*-S10H3 expression is maintained. (Scale bar, 200μm). Dotted and dashed lines indicate the surface of the spinal cord and the white-grey matter border, respectively.

**Supplementary Figure 3**


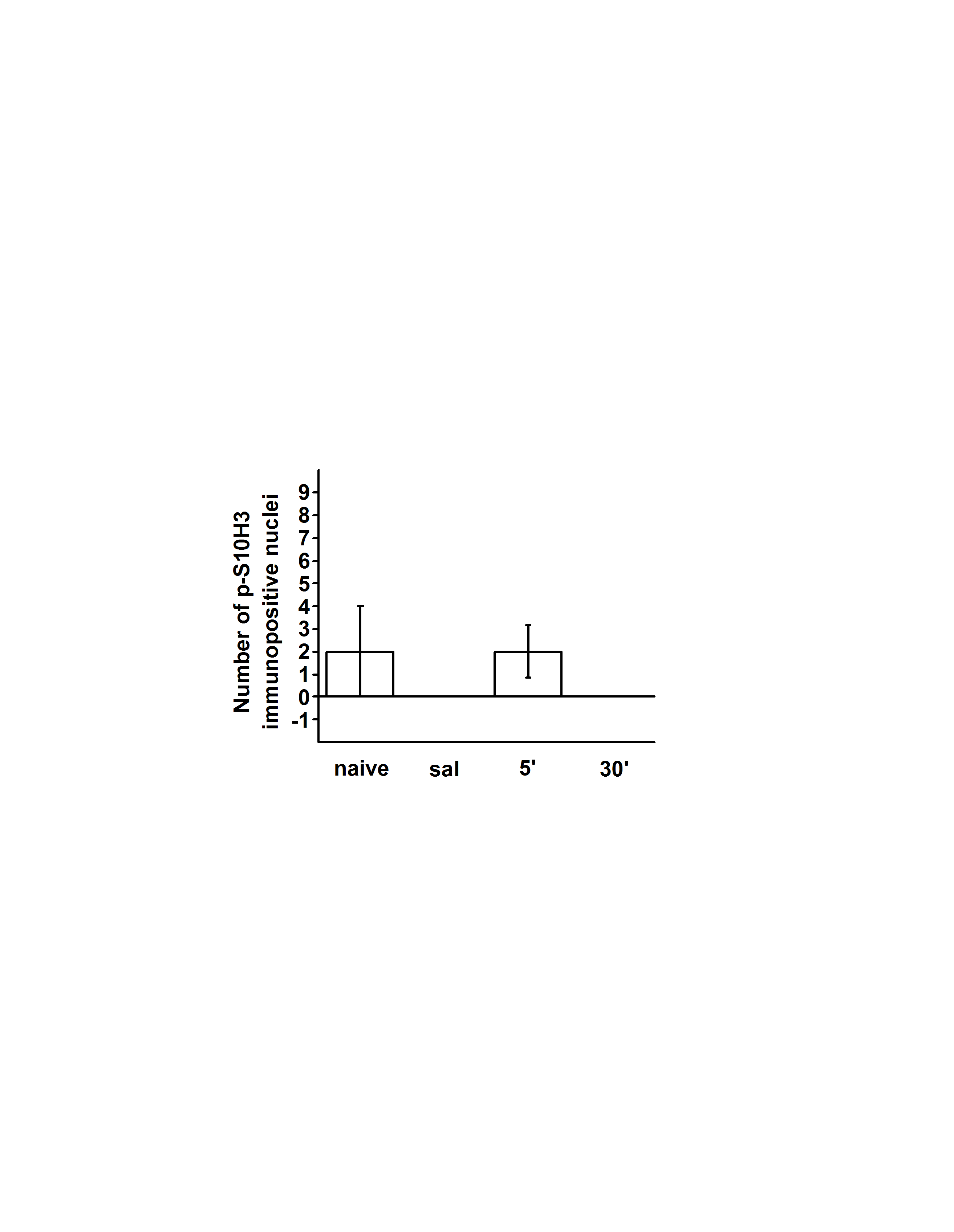


Number of p-S10H3 immunopositive nuclei in contralateral side of the superficial spinal dorsal horn of naive (naive), saline-injected (sal) and capsaicin-injected rat 5 minutes (5’) and 30 minutes (30’) after the injection. Neither saline injection nor capsaicin injection induces up-regulation in p-S10H3 expression in the contralateral side.

**Figure 4**

**
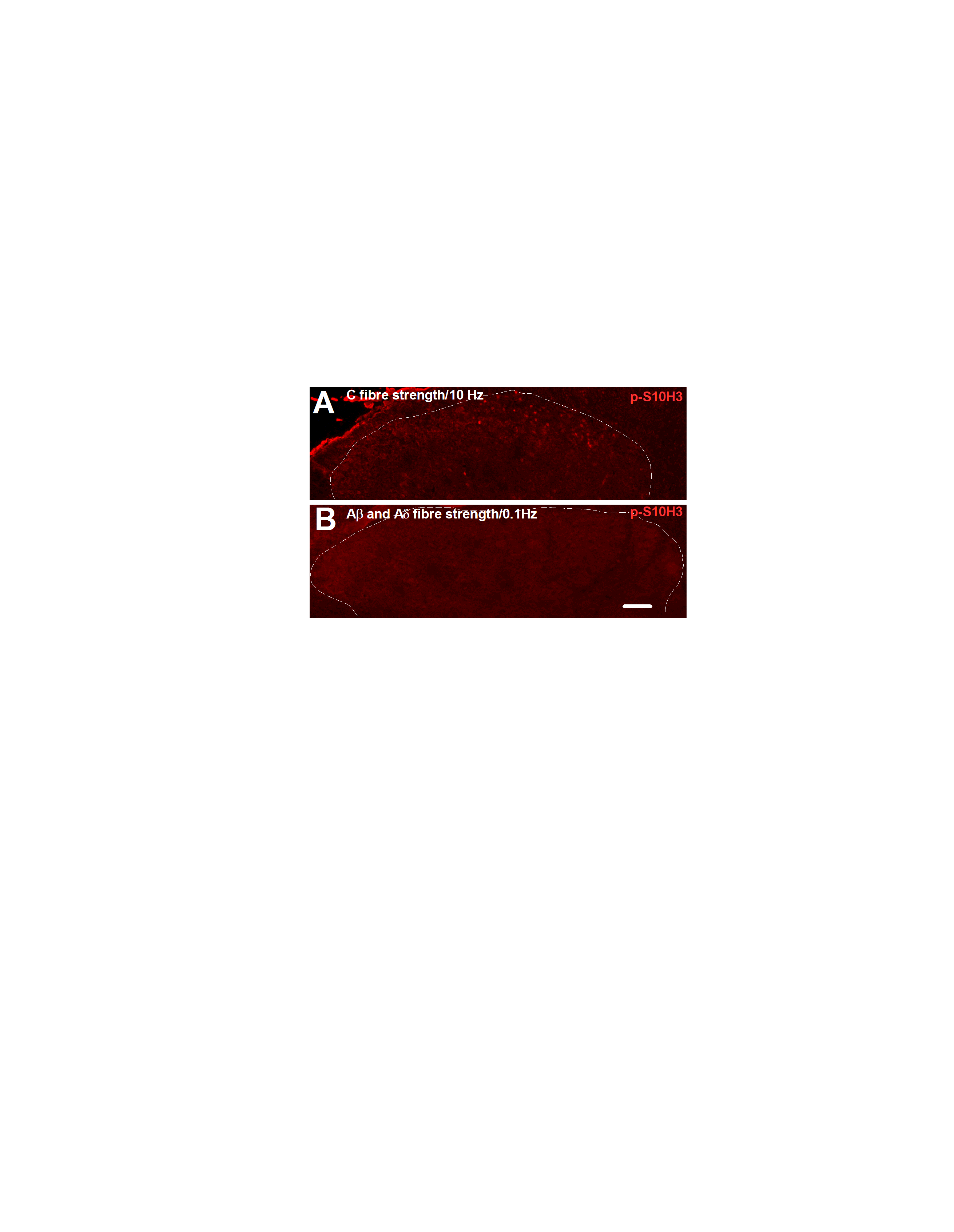
**

Images of sections cut from the ipsilatreal side of the L4 spinal segment after electrical stimulation of the femoral nerve.

High frequency repetitive electrical stimulation with C fibre strength (A), but not low frequency stimulation with Aβ and Aδ fibre strength (B), of the femoral nerve up-regulates *p*-S10H3 expression in a group of nuclei in the superficial dorsal horn. Scale bar= 100μm.

The dashed lines indicate the white-grey matter border on both images.

**Supplementary Figure 5**


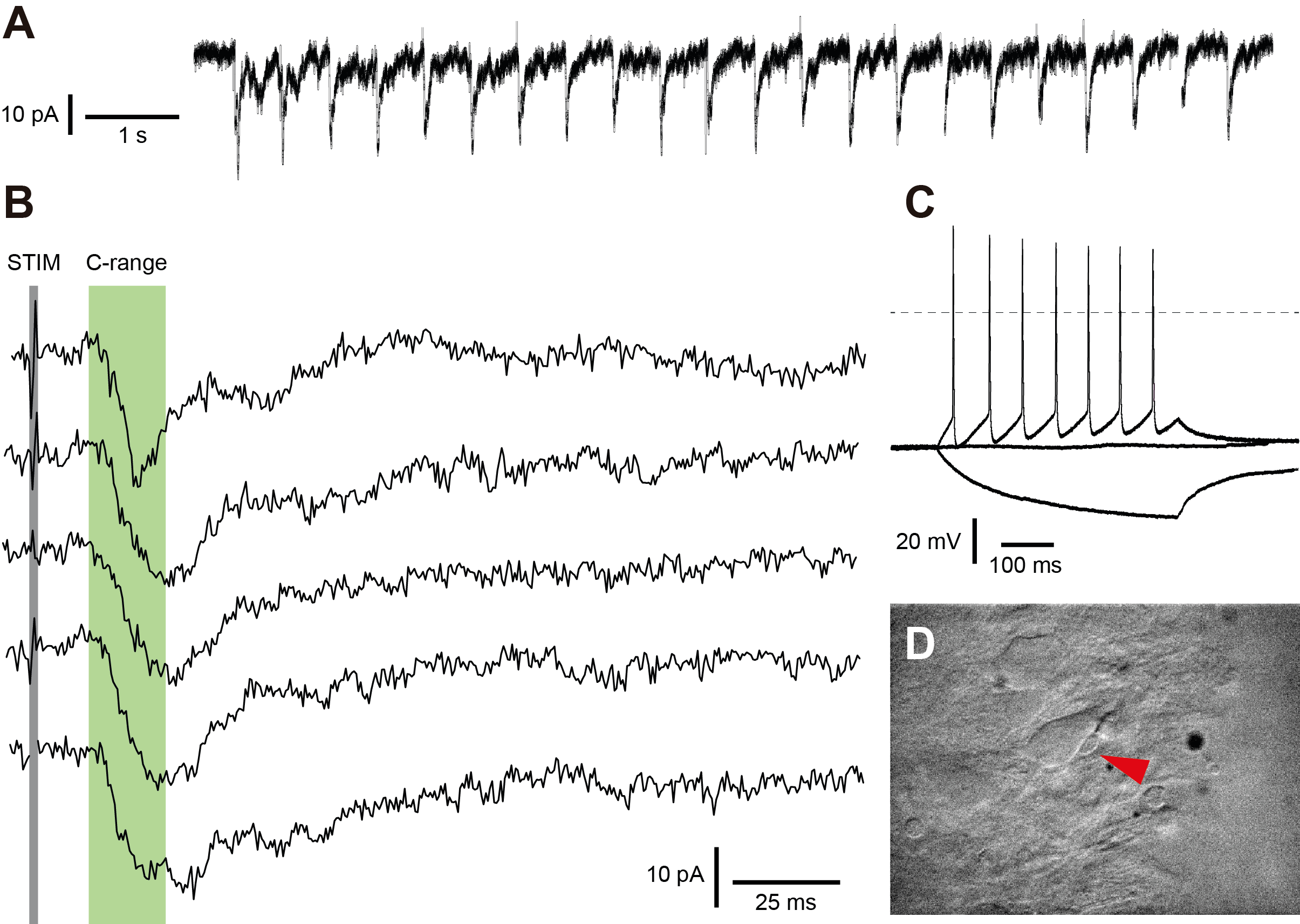


The effectiveness of dorsal root electrical stimulation was tested by monitoring primary afferent evoked currents of superficial dorsal horn neurons (n=4). Whole-cell patch-clamp recordings were performed at room temperature using an EPC 10 Double amplifier (HEKA, Germany). Patch pipettes with 6-8 MΩ pipette resistance were filled with a solution containing (in mM): K-gluconate, 120; NaCl, 5; 4-(2-hydroxyethyl)-1- piperazineethanesulfonic acid (HEPES), 10; EGTA, 2; CaCl2, 0.1; Mg-ATP, 5; Na3-GTP, 0.3; Na2- phosphocreatinine, 10; biocytin, 8; pH 7.3.

(A) Repetitive dorsal root stimulation evoked reliable excitatory postsynaptic currents (EPSCs) in the recorded neurons.

(B) The consecutive EPSCs showed a delay that was compatible with C-fiber mediated postsynaptic current latencies (green highlight indicates the C-fiber latency range while the grey line highlights the stimulation artefact).

(C) The particular lamina I neuron receiving the primary afferent input shown on panels (A) and (B) responded with a tonic firing pattern to suprathreshold depolarizing current steps.

(D) The neuron, the responses of which are shown in (A)-(C) had a triangular soma located on the surface of the dorsal horn.

**Supplementary Figure 6**

**
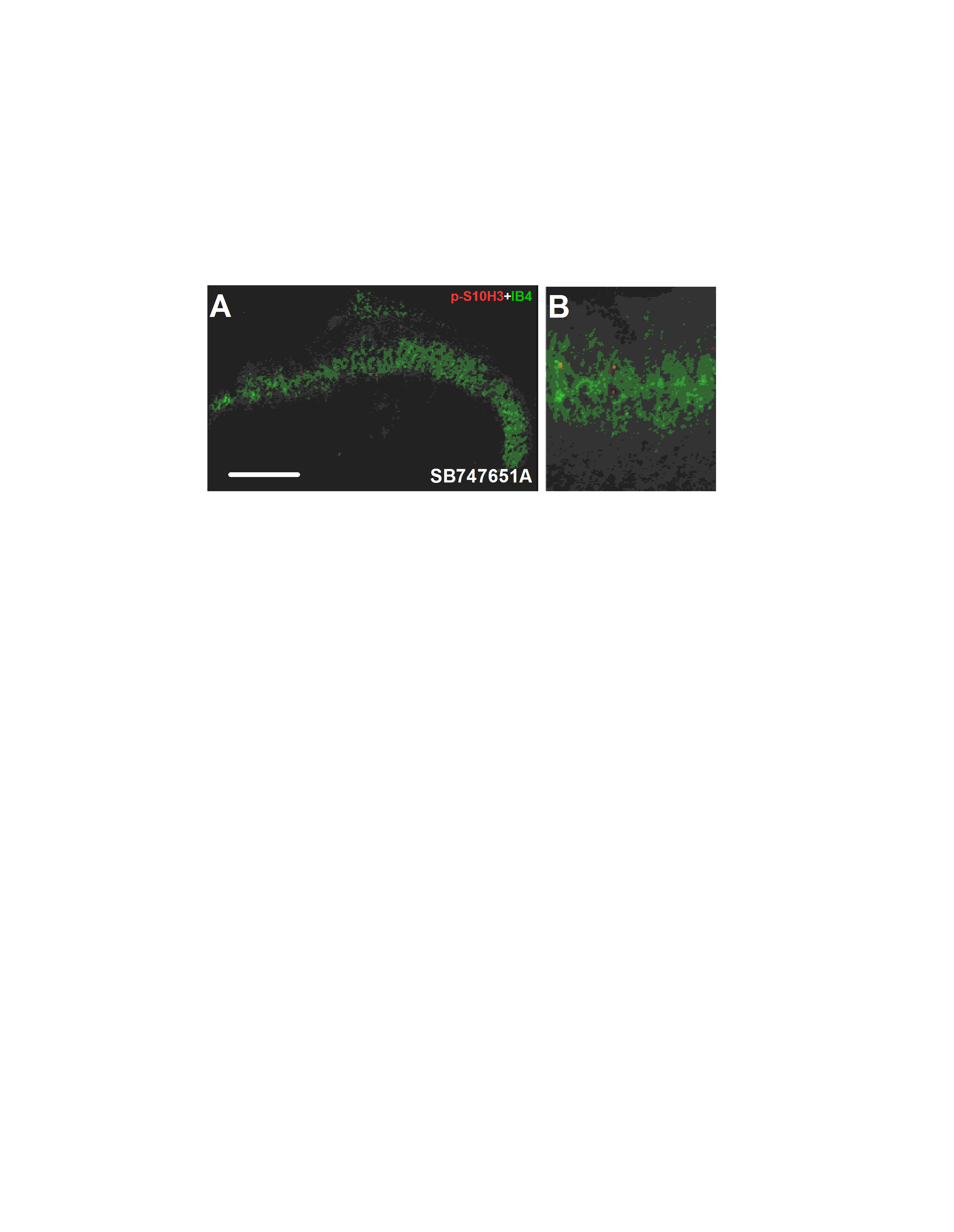
**

(A) and (B) Images of a section cut from slices prepared from the L4-L5 spinal segments and exposed to 10μM SB 747651A followed by immunostaining with an anti-*p*-S10H3 antibody. IB4 labelling was also performed to label the ventral border of lamina II_i_. (Scale bar, 200μm and 100μm, respectively on (A) and (B).)

**Supplementary Figure 7**


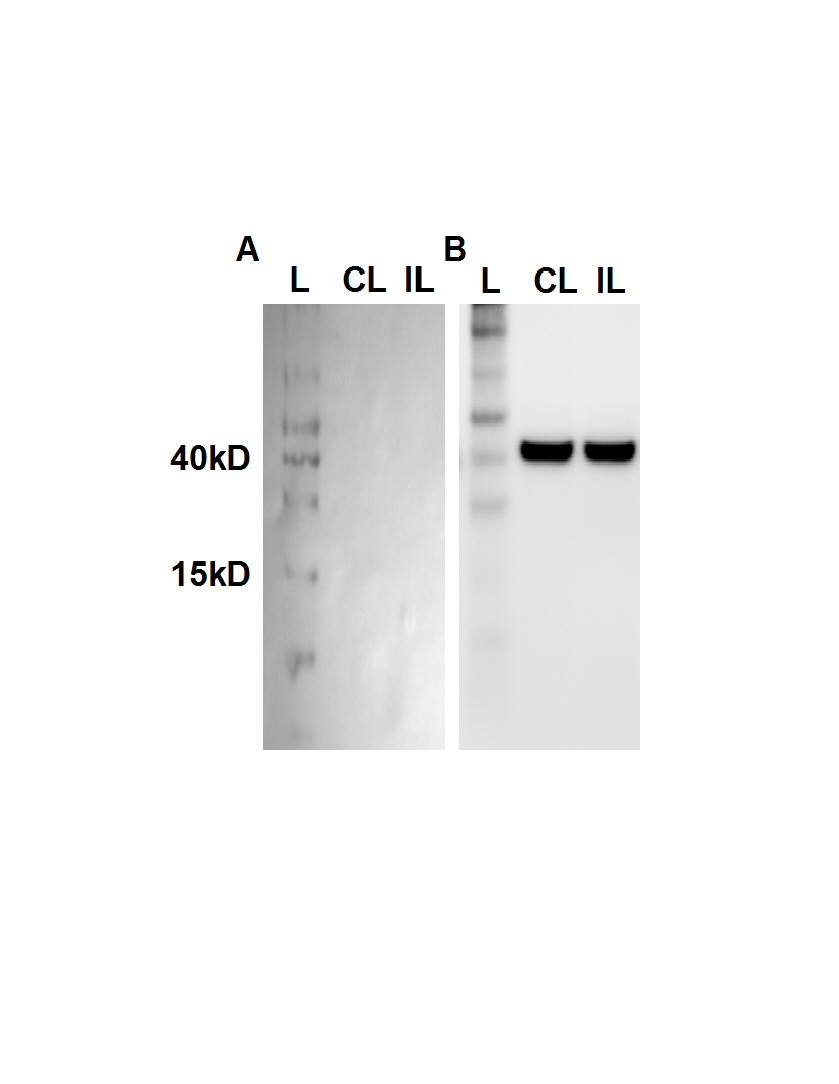


Images of control immunoblots. (A) shows the lack of recognised protein, in a sample extracted from a spinal cord 5 minutes after capsaicin injection into the paw, when both the anti-*p*-S10H3 and anti-β-actin antibodies were replaced with non-immune IgG. (B) shows the lack of recognised *p*-S10H3 (~17kD), in a sample extracted from a spinal cord 5 minutes after burn injury of the paw, when the anti-*p*-S10H3 was replaced with non-immune IgG. In this experiment, the ant-β-actin antibody was used to show the protein loading.

**Supplementary Figure 8.**


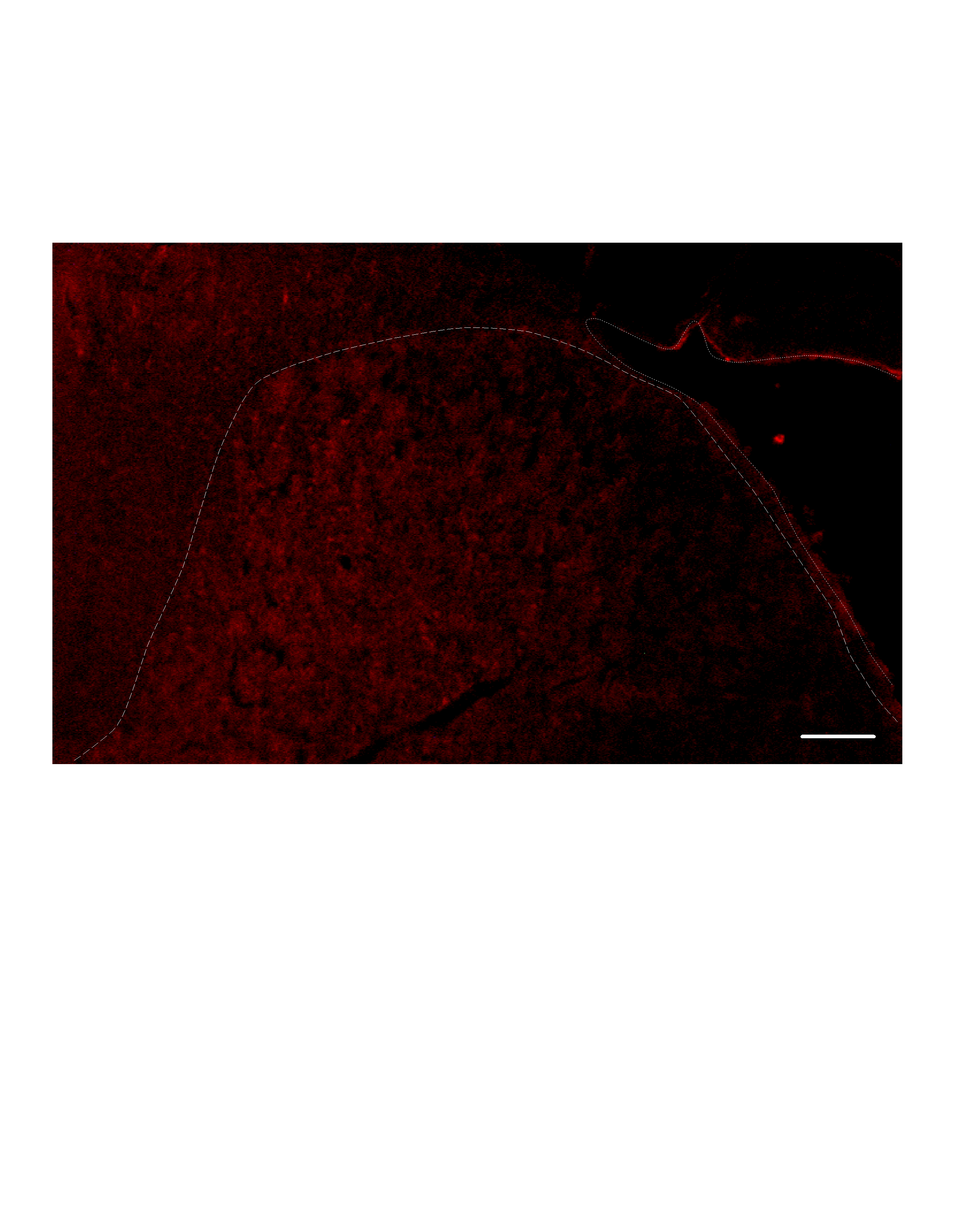


An image of a section cut from the ipsilatreal side of the L4-L5 spinal segments of a rat 5 minutes after burn injury. During the incubation of this section, the anti-p-S10H3 antibody was replaced by normal serum. The secondary antibody was applied as usual. The dotted and dashed line indicates the surface of the spinal cord and the white-grey matter border, respectively. (Scale bar, 100μm.)
