# Supplementary material for: Phosphorylated Histone 3 at Serine 10 Identifies Activated Spinal Neurons and Contributes to the Development of Tissue Injury-Associated Pain
Source: Sci Rep. 2017 Jan 25;7:41221. doi: 10.1038/srep41221 (PMC5264160; doi:10.1038/srep41221)
Supplement: Supplementary Dataset [file srep41221-s2.doc]

**SUPPLEMENTARY TABLES**

**PHOSPHORYLATED HISTONE 3 AT SERINE 10 IDENTIFIES ACTIVATED SPINAL NEURONS AND CONTRIBUTES TO THE DEVELOPMENT OF TISSUE INJURY-ASSOCIATED PAIN**

Jose Vicente Torres-Pérez^1^, Péter Sántha^2^, Angelika Varga^3,4^, Peter Szucs^3,4^, Joao Sousa-Valente^1^; Botond Gaal^4^, Miklós Sivadó^3^, Anna P Andreou^1^, Sara Beattie^1^, Bence Nagy^5^, Klara Matesz^4^, J. Simon C. Arthur^6^, Gábor Jancsó^2^ and Istvan Nagy^1$^

^1^Nociception Group, Section of Anaesthetics, Pain Medicine and Intensive Care, Department of Surgery and Cancer, Imperial College London, London, SW10 9NH, United Kingdom; ^2^Department of Physiology, University of Szeged, Szeged, H-6720, Hungary; ^3^MTA-DE-NAP B-Pain Control Research Group, Department of Anatomy, Histology and Embryology and Department of Physiology, University of Debrecen, Debrecen, H-4012, Hungary; ^4^Department of Anatomy, Histology and Embryology, University of Debrecen, Debrecen, H-4012, Hungary; ^5^The Ipswich Hospital, Ipswich, IP4 5PD, United Kingdom; ^6^Division of Cell Signalling and Immunology, College of Life Sciences, Sir James Black Centre, University of Dundee, Dundee DD1 5EH, United Kingdom

Running title: p-S10H3 in spinal nociceptive processing

**Supplementary Table 1.**

**List of primary antibodies**

| **Primary antibodies** | | | | | | | | | | |
| --- | --- | --- | --- | --- | --- | --- | --- | --- | --- | --- |
| **Target** | **Company** | **Catalogue Number** | **Host Species** | **Staining Protocol** | **Concentration used** | **Western Blot** | **Clonality** | **Immunogen** | **Company's control** | **Our control** |
| **Calbindin D-28k** | Swant | 300 | mouse | IF | 1:1,000 | ✗ | m | Calbindin D-28k purified from chicken gut | no staining in the brain of calbindin D-28k knock out mice | - |
| **c-Fos** | Santa Cruz | sc-52 | rabbit | IF | 1:1,000 | ✗ | p | Around 15-25 aa, located between the first 50 aa of the N-terminus of c-Fos of human origin* | - | - |
| **GFAP** | Synaptic Systems | 173004 | guinea pig | IF | 1:500 | ✗ | p | Recombinant full length human GFAP | mouse hippocampus | - |
| **biotin-conjugated isolectin B4 (IB4)** | Sigma | L2140 | *B. simplicifolia* | - | 1:1,000 | ✗ | - | - | - | - |
| **IBA 1** | Synaptic sistems | 234004 | guinea pig | IF | 1:500 | ✗ | p | aa 134 - 147 rat IBA 1 | mouse brain sections | - |
| **MAP2** | Abcam | ab5392 | chicken | IF | 1:5,000 | ✗ | p | Full length cow brain MAP2 purified from the material that pellets with GTP polymerized tubulin | cultured rat cortical neurons | - |
| **NeuN** | Millipore | mab377 | mouse | IF | 1:500-2,000 | ✗ | m | Purified cell nuclei from mouse brain | Positive control -Brain Tissue. | - |
| **OX42 (CD11b)** | ABDserotec | MCA275R | mouse | IF | 1:1,000 | ✗ | m | whole resident rat peritoneal macrophages | Clone validated on Rat Peritoneal Macrophages by flow Cytometry | - |
| **pan-histone H3 CT** | Millipore | 07-690 | rabbit | ✗ | - | ✓ 1:4,000 | p | KLH-conjugated, synthetic peptide corresponding to the C-terminus of human Histone H3 | Immunoblot:acid extracted proteins from untreated, sodium butyrate or colcemid treated HeLa cells. | Embrionic tissues with high mitotic factor and blocking peptide provided by the company |
| **parvalbumin** | Swant | PV-235 | mouse | IF | 1:2,000 | ✗ | m | Parvalbumin purified from carp muscles | no staining the brain of parvalbumin knock out mice | - |
| **pERK1/2** | Neuromics | RA15002 | rabbit | IF | 1:1,500 | ✗ | p | Synthetic phosphopeptide corresponding to residues surrounding T202/Y204 of human, rat and mouse ERK* | Validated by Western Blot (lysates of HeLa human cervical epithelial carcinoma cell line untreated or treated with 200 nM PMA) and IHC (rat brain; cortex) | - |
| **pS10H3** | Santa Cruz | sc-8656-R | rabbit | TSA/DAB | 1:200-750 | ✓ 1:200-500 | p | Around 15-25 aa, located between the first 50 aa of the N-terminus of pS10H3 of human origin* | Blocking peptide provided and HeLa + Calyculin A whole cell lysates | Embrionic tissues with high mitotic factor and blocking peptide provided by the company |
| **TRPV1** | Neuromics | GP141000 | guinea pig | IF | 1:500-1,500 | ✗ | p | YTGSLKPEDAEVFKDSMVPGEK; C terminal aa of rat VR1 | Immunohistochemistry on rat dorsal root ganglion (PFA perfusion fizzed  frozen sections at a dilution of 1/1000) | - |
| **β actin** | Millipore | mab1522 | mouse | ✗ | - | ✓ 1:2,000 | m | Chicken gizzard actin; smooth muscle gamma & alpha actin | Actin from Chicken, Rabbit, Rat, Monkey, or Human | - |
| *** company does not disclose sequence** | | | | | | | | | | |

**Supplementary Table 2.**

**List of secondary antibodies used and specific characteristics for each one**.

| **Secondary antibodies** | | | | | | |
| --- | --- | --- | --- | --- | --- | --- |
| **Target/fluorochrome** | **Company** | **Catalogue number** | **Host** | **Staining Protocol** | **Concentration for staining** | **Western Blot** |
| **Chicken Alexa Fluor 594** | Molecular Probes | A11042 | goat | IF | 1:1,000 | ✗ |
| **FITC-avidin conjugate** | Jackson Immuno Research | SA-AF488 | -n/a | IF | 1:200 | ✗ |
| **Goat Alexa Fluor 488** | Molecular Probes | A-11055 | donkey | IF | 1:1,000 | ✗ |
| **Guinea pig Alexa Fluor 488** | Molecular Probes | A11075 | donkey | IF | 1:1,000 | ✗ |
| **Guinea pig Alexa Fluor 647** | Molecular Probes | A21450 | goat | IF | 1:1,000 | ✗ |
| **Biotin HRP-linked** | Cell Signalling | 7075 | goat | ✗ | ✗ | ✓ 1:1,000 |
| **Mouse Alexa Fluor 488** | Life Technologies | A11001 | goat | IF | 1:1,000 | ✗ |
| **Mouse Alexa Fluor 488** | Molecular Probes | A21202 | donkey | IF | 1:1,000 | ✗ |
| **Mouse Alexa Fluor 568** | Molecular Probes | A10037 | donkey | IF | 1:1,000 | ✗ |
| **Mouse HRP-conjugated** | Cell Signaling | 7076S | horse | ✗ | ✗ | ✓ 1:1,000 |
| **Rabbit Alexa Fluor 488** | Molecular Probes | A21206 | donkey | IF | 1:1,000 | ✗ |
| **Rabbit Alexa Fluor 555** | Life Technologies | A21428 | goat | IF | 1:1,000 | ✗ |
| **Rabbit Alexa Fluor 568** | Molecular Probes | A10042 | donkey | IF | 1:1,000 | ✗ |
| **Rabbit biotin-SP-conjugated** | Jackson Immuno Research | 711-066-152 | donkey | TSA/DAB | 1:500 | ✗ |
| **Rabbit HRP-conjugated** | Cell Signaling | 7074S | goat | ✗ | ✗ | ✓ 1:1,000 |
| **Rabbit HRP-conjugated** | Santa Cruz | Sc2313 | donkey | ✗ | ✗ | ✓ 1:5,000 |
| **Streptavidin Alexa Fluor 488** | Invitrogen | S-11223 | - | TSA | 1:1,000 | ✗ |
| **Streptavidin Alexa Fluor 546** | Invitrogen | S-11225 | - | TSA | 1:1,000 | ✗ |
